# Biomarkers of Fumonisin Exposure in Pigs Fed the Maximum Recommended Level in Europe

**DOI:** 10.3390/toxins17020069

**Published:** 2025-02-04

**Authors:** Elodie Lassallette, Alix Pierron, Didier Tardieu, Solène Reymondaud, Marie Gallissot, Maria Angeles Rodriguez, Pi Nyvall Collén, Olivier Roy, Philippe Guerre

**Affiliations:** 1 National Veterinary School of Toulouse, ENVT, Université de Toulouse, 31076 Toulouse, France; elodie.lassallette@envt.fr (E.L.); alix.pierron@envt.fr (A.P.); didier.tardieu@envt.fr (D.T.); solene.reymondaud@univ-tlse3.fr (S.R.); 2 Olmix S.A., ZA du Haut du Bois, 56580 Bréhan, France; m.gallissot@olmix.com (M.G.); mrodriguez@olmix.com (M.A.R.); pnyvall@olmix.com (P.N.C.); 3 Cebiphar, 1 Rue de la Bodinière, 37230 Fondettes, France; oroy@cebiphar.com

**Keywords:** fumonisins, sphingolipids, liver, kidney, lung, plasma, pig, AlgoClay, ratio, biomarker

## Abstract

This study investigated biomarkers of fumonisin exposure in pigs fed diets contaminated with fumonisins at the European Union’s maximum recommended level. Pigs were assigned to either a fumonisin (FB) diet or a fumonisin plus AlgoClay (FB + AC) diet for durations of 4, 9, and 14 days. At 14 days, the plasma Sa1P:So1P ratio increased in pigs fed the FB diet, while the Sa:So ratio remained unchanged. In the liver, FB1 was detected at four days of exposure, with the concentration tending to increase through day 14. The Sa:So and C22-24:C16 ratios of 18:1-, 18:2-, and m18:1-ceramides were elevated at 9 and 14 days, respectively. In the kidneys, FB1 was only detectable at 14 days, and the Sa:So and C22-24:C16 ratios of 18:1-ceramides were increased. In both the liver and kidneys, the increase in the C22-24:C16 ratio was attributed to a reduction of C16 ceramides. In the lungs, no FB1 was detected; however, the Sa:So and Sa1P:So1P ratios increased, and C16 ceramide concentrations decreased at 14 days. Feeding the pigs the FB + AC diet resulted in a reduction of the FB1 tissue-to-feed ratio in the liver and kidneys but did not affect the Sa:So or Sa1P:So1P ratios. Interestingly, the decreases in C16 ceramides observed in the FB diet group were no longer detectable in the FB + AC group. Overall, these findings highlight the complexity of the relationship between FB1 tissue concentrations and sphingolipid changes, suggesting that a comprehensive analysis of multiple biomarkers is required to fully understand fumonisin’s effects.

## 1. Introduction

The characterization of biomarkers is a critical health issue, particularly in the context of contaminant exposure [1]. Biomarkers are generally categorized into two types: exposure biomarkers and effect biomarkers. Exposure biomarkers indicate the direct dose of a contaminant or its metabolites in fluids and tissues, while effect biomarkers identify biological effects specific enough to signify exposure to a contaminant. These effects can range from adaptive, with no toxicological significance, to adverse, reflecting a contaminant’s harmful impact on health. These biomarkers can be measured in plasma or urine, the latter being particularly used in humans to monitor exposure to mycotoxins [2,3,4,5]. Various monitoring programmes are also carried out on meat products from farmed animals to correlate any changes in performance or lesions with mycotoxin levels in tissues and to estimate the risks to human consumers associated with the consumption of mycotoxin-contaminated meat products [6,7,8,9].

Fumonisins, a group of mycotoxins predominantly produced by fungi of the genus *Fusarium*, notably *Fusarium verticillioides* and *Fusarium proliferatum*, are commonly found in cereals, especially maize [10,11,12]. Their prevalence is highest in tropical and subtropical regions, where favorable climatic and storage conditions promote fungal growth [13,14]. Among fumonisins, fumonisin B1 (FB1) is the most abundant and toxic variant [10]. Fumonisin toxicity manifests differently across species. In animals, it has been linked to conditions such as equine leukoencephalomalacia in horses and pulmonary edema in pigs [13,14]. In humans, fumonisins have been associated with growth retardation and esophageal cancer [13,14], prompting their classification by the International Agency for Research on Cancer (IARC) as possibly carcinogenic (Group 2B). Regulatory bodies have established a tolerable maximum exposure dose of 2 μg FB1 + FB2 + FB3/kg body weight for humans [15] and have recommended limits for fumonisin concentrations in animal feed [10,16].

The toxic effects of fumonisins vary significantly depending on the species and exposure duration, with toxic effects often being bioaccumulative [13,14]. Earlier studies using radiolabeled toxins indicated that fumonisins bioaccumulate [17]. However, subsequent research using non-radiolabeled toxins suggested the rapid elimination of FB1 [18,19]. Recent advances in mass spectrometry have confirmed the bioaccumulative potential of FB1 in poultry, particularly when exposed to the recommended maximum dietary level of 20 mg FB1 + FB2/kg [20]. Furthermore, studies conducted at a lower concentration of 7 mg FB1 + FB2/kg reported a hepatic half-life of approximately 60 h for FB1 in chickens and turkeys [21]. These findings underscore the utility of the tissue FB1 as a reliable biomarker for fumonisin exposure in these species.

The toxic effects of fumonisin are diverse, but numerous studies have identified a primary mechanism tied to disruptions in sphingolipid metabolism. This disruption arises from the structural similarity between FB1 and sphingoid bases [22], which leads to an early increase in cellular sphinganine (Sa), often followed by a delayed and variable rise in sphingosine (So). Consequently, the Sa:So ratio has been widely adopted as a biomarker for fumonisin effects across various animal species. Its sensitivity allows it to detect changes influenced by both the dietary concentration of fumonisin and the duration of exposure [22,23,24]. Beyond the Sa:So ratio, alternative biomarkers have been proposed to enhance sensitivity and specificity. For example, the Sa1P:So1P ratio, which is based on phosphorylated sphingoid bases, has demonstrated superior sensitivity in plasma analyses for mice, humans, and chickens [25,26,27,28]. More recently, targeted sphingolipid analysis in chickens and turkeys has introduced the C22-24:C16 ratio as another promising biomarker [25,29]. This ratio appears to be at least as sensitive as the Sa:So ratio in chickens and, importantly, less influenced by exposure duration [29].

Multiple studies have validated the Sa:So ratio as an effective biomarker for detecting fumonisin exposure in pigs [23,24,30,31,32,33,34,35,36]. However, these studies also emphasize the complexity of fumonisin’s impact on this biomarker. For instance, different studies conducted at high doses of fumonisins in feed have shown that alterations in Sa levels and the Sa:So ratio across the liver, kidney, and lung were not directly related to the organ-specific toxicity of fumonisin in pigs [23,24]. Further studies in mice with myriocin, an inhibitor of serine palmitoyltransferase, demonstrated that myriocin alone was not hepatotoxic, and the combination of myriocin plus FB1 completely prevented the FB1-induced elevation of hepatic sphinganine but did not prevent the hepatotoxicity [37]. Other studies of pigs at relatively low doses of fumonisins in feed reported changes in the Sa:So ratio in the plasma of pigs consuming diets with 3.7 mg FB1 + FB2/kg for 28 days, while higher concentrations of 8.1 and 12.2 mg FB1 + FB2/kg showed no effect [33]. Despite extensive research on fumonisin toxicity and the important role of complex sphingolipids in its toxicity [22,37], only one study has explored fumonisin effects on the sphingolipidome in pigs [31]. This study involved the oral administration of 2 mg FB1/kg of body weight, corresponding to dietary levels of 20 to 25 mg FB1/kg. It revealed variable effects on sphingolipid profiles in the liver and lung, yet tissue FB1 levels were not measured [31]. Furthermore, no studies have yet examined the influence of low-dose fumonisin exposure on both tissue FB1 concentrations and the sphingolipidome in pigs.

This study aims to address these gaps by investigating the effects of fumonisins at the European recommended maximum levels on various biomarkers of fumonisin exposure and toxicity in pigs. Tissue FB1 concentrations were assessed as exposure biomarkers, while Sa:So, Sa1P:So1P, and C22-24:C16 ratios were evaluated as effect biomarkers in the liver, kidney, and lung. Effect biomarkers alone were measured in plasma. To investigate the early presence and effects of fumonisin and to assess whether its accumulation and toxic effects increase with prolonged exposure, this study was conducted using pigs fed fumonisin-contaminated diets for 4, 9, and 14 days. The dose and the duration of the exposure were chosen to be representative of the situations that are likely to occur on pig farms [38]. Additionally, the study examined the impact of diets containing AlgoClay, both alone and in combination with fumonisins. AlgoClay, a known toxin-binder, has previously been shown to reduce FB1 concentrations in the liver and muscle of chickens by 40% and 50%, respectively [20]. This research aimed to determine if AlgoClay produces similar reductions in tissue FB1 concentrations in pigs. Another critical objective was to explore whether changes in tissue fumonisin levels induced by AlgoClay correspond with alterations in the sphingolipidome.

## 2. Results

### 2.1. Effect on Performance

The experimental diets included a control diet (Con), a diet containing AlgoClay (AC) at 450 mg/kg, a diet with fumonisins (FB) at 5.29 mg FB1 + FB2/kg, and a combined diet with fumonisins and AlgoClay (FB + AC) at 5.75 mg FB1 + FB2/kg and 450 mg AlgoClay/kg. These diets were administered over 4 to 14 days. No mortality or clinical signs were observed in any of the animals during the study period.

Animal weights at the start of the experiment (D1) and following 7 (D7) and 13 (D13) days of diet exposure are presented in Table 1, along with average feed consumption per group for the duration of the study (days 1 to 14). Additionally, average and relative liver and kidney weights were recorded and are summarized in Table 1, whereas individual liver and kidney weights are shown in Figure S1. The data revealed no significant differences between the groups for any variable, except for a change in relative liver weight. However, this change was not significantly different from the control group and showed no link to the duration of exposure.

### 2.2. Effect on Plasma Biochemistry

Table 2 presents the concentrations of total protein, albumin, cholesterol, triglycerides, urea, creatinine, and the activities of LDH, ASAT, ALAT, PAL, and CPK measured in the plasma. Significant differences between groups were observed for several of these variables, with no clear effect attributed to the duration of exposure to fumonisins or AlgoClay. Among the significant changes, pigs fed with the FB diet for nine days exhibited increased concentrations of albumin and cholesterol. However, after 14 days of exposure, these values returned to levels comparable to the control group. In contrast, the FB + AC group showed significantly higher levels of creatinine, urea, and CPK after nine days, but these variables were not significantly different from the control group after 14 days of exposure.

### 2.3. Effect on Sphingolipids in the Liver

**Figure 1:**
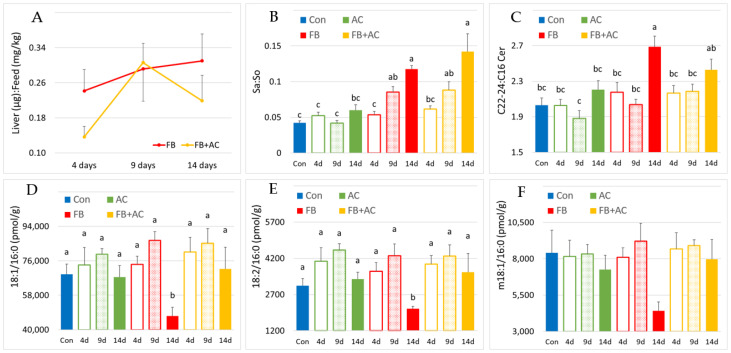
Effects in pig livers of diets containing fumonisins (FB) and AlgoClay (AC) alone or in mixtures (AC + FB) for 4, 9, and 14 days on different biomarkers of fumonisin exposure and on concentrations of 18:1-, 18:2-, and m18:1-C16-ceramides. Con: control; Sa: sphinganine; So: sphingosine; Cer: ceramides; d = day. (**A**) Liver:Feed ratio in the liver as a function of exposure time for FB and AC + FB groups. (**B**) Sa:So ratio in the liver. (**C**) Ratio of C22-24:C16 for ceramides in the liver. Concentration in pmol/g of the liver of (**D**) 18:1/16:0, (**E**) 18:2/16:0, and (**F**) m18:1/16:0. Results are expressed as mean with standard error, n = 5 for Con and AC alone groups, and n = 6 for FB alone and AC + FB groups. ANOVA was used to estimate differences between groups. Statistically different groups are indicated by different letters (*p* < 0.05).

In the liver, FB1 was detectable as early as four days after the start of feeding the FB and FB + AC diets, with concentrations increasing as the duration of exposure extended in pigs fed the FB diet (Table 3). The Liver:Feed ratio, measured at 14 days of exposure, was 28% higher in pigs that were fed the FB diet compared to those fed the FB diet for only four days, though this difference was not statistically significant. At 9 days of exposure, the Liver:Feed ratios of pigs on the FB diet and those on the FB + AC diet were identical. However, after 4 and 14 days, pigs fed with the FB + AC diet showed 43% and 29% lower Liver:Feed ratios, respectively, compared to those fed the FB diet, but these differences were also not statistically significant (Figure 1A).

Sphingolipid concentrations in the liver of pigs are presented in Table S1. The Sa:So ratio in liver tissue increased with the duration of exposure to fumonisins in pigs fed both the FB and FB + AC diets (Figure 1B). The difference from unexposed controls became significant after nine days of exposure, with the rise in the Sa:So ratio largely attributed to an increase in Sa (Table 3). Other sphingoid bases, such as phytosphingosine (t18:0), sphingadienine (d18:2), deoxysphingosine (m18:1), deoxysphinganine (m18: 0), deoxysphingosine (m18:1), deoxymethylsphingosine (m17:1), and sphinganine and sphingosine in the d20:0 and d20:1 forms, did not show significant differences between groups, indicating that their concentrations were not notably affected by the duration of exposure to fumonisins or AlgoClay.

The C22-24:C16 ratios for various SL classes are presented in Table 3, with the C22-24:C16 ratio for d18:1-ceramides shown in Figure 1C. A significant difference from the control group, representing a 32% increase in the ratio, was observed only in pigs fed the FB diet for 14 days. In contrast, at 14 days of exposure, the C22-24:C16 ratio in pigs fed the FB + AC diet did not differ from that of the control group. At 14 days of exposure to the FB diet, the impact of fumonisins on the C22-24:C16 ratio was primarily due to a significant 36% decrease in the 18:1/16:0 concentrations (Figure 1D). However, the 18:1/16:0 concentrations in the liver of pigs fed with the FB + AC diet were not significantly different from those in pigs fed the control diet. Similar results were observed when 18:1/16:0 concentrations were expressed as a percentage of total ceramides (Figure S2B). The effects of fumonisins on C22-24 ceramide concentrations varied depending on the method of expression. When expressed as liver concentrations, the effect of fumonisins was not significant. However, when the results were expressed as percentages of total ceramides, a significant increase was noted (Figure S2C,D). The effects of fumonisins on the C22-24:C16 ratios for other sphingolipid classes, including dihydroceramides, sphingomyelins, dihydrosphingomyelins, monohexosylceramides, and lactosylceramides, showed similar trends, though the effects were generally less pronounced than those observed for 18:1-ceramides (Table S1).

Similarly, the distribution of the FB diet for 14 days resulted in a 31% decrease in 18:2/16:0 concentrations and a 46% decrease in m18:1/16:0 concentrations (Figure 1E,F), while no effect was observed on t18:0/16:0 concentrations (Table S1). This effect was accompanied by an increase in the C22-24:C16 ratio measured for m18:1-ceramides in pigs fed the FB diet for 14 days (Table S1), although the C22-24:C16 ratio for d18:2-ceramides remained unaffected. As observed with 18:1-ceramides, administration of the FB + AC diet prevented the effects of fumonisins on the 18:2/16:0 and m18:1/16:0 concentrations (Figure 1E,F) and on the C22-24:C16 ratio measured for m18:1-ceramides (Table S1).

**Figure 2:**
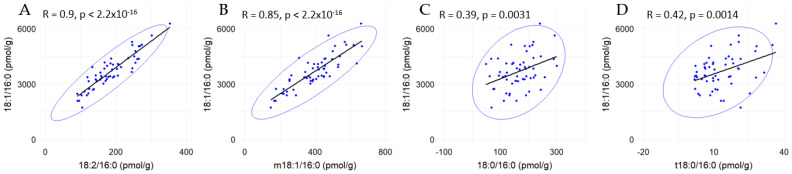
Correlations between C16 ceramides and different sphingoid bases. Correlation lines and confidence ellipses (0.99) were obtained from 56 pigs fed a control diet and diets containing fumonisins and AlgoClay alone or mixed for 4, 9, and 14 days between 18:1/16:0 and (**A**) 18:2/16:0, (**B**) m18:1/16:0, (**C**) 18:0/16:0, and (**D**) t18:0/16:0.

Because fumonisins decrease the concentrations of 18:1-, 18:2-, and m18:1-C16-ceramides but have no effect on 18:0- and t18:0-C16-ceramide levels, the correlations between these analytes were analyzed (Figure 2). These results revealed a very strong correlation between 18:1/16:0 and 18:2/16:0, with an R value of 0.9 (Figure 2A), indicating a robust connection between these two ceramide species. Similarly, a strong correlation was observed between 18:1/16:0 and m18:1/16:0, with an R value of 0.85 (Figure 2B), as well as between 18:2/16:0 and m18:1/16:0 (R = 0.73) (Figure S3A). In contrast, the correlation between 18:1/16:0 and 18:0/16:0 was much weaker, with an R value of 0.39 (Figure 2C), and similarly weak correlations were found between 18:1/16:0 and t18:0/16:0 (R = 0.42) (Figure 2D). No significant correlation was observed between 18:0/16:0 and t18:0/16:0, with an R value of only 0.07 (Figure S3B).

### 2.4. Effect on Sphingolipids in the Kidney

**Figure 3:**
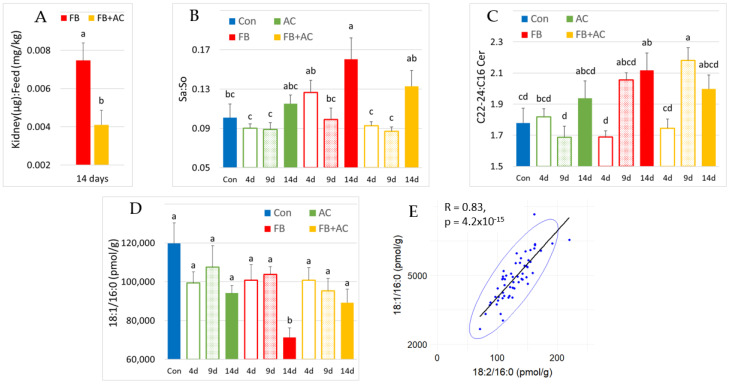
Effects on pig kidneys of feeding diets containing fumonisins (FB) and AlgoClay (AC) alone or in mixture (AC + FB) for 4, 9, and 14 days on different biomarkers of fumonisin exposure and on the concentration of 18:1-C16-ceramides. Con: control; Sa: sphinganine; So: sphingosine; Cer: ceramides; d = day. (**A**) Kidney:Feed ratio in kidneys as a function of exposure time for FB and AC + FB groups. (**B**) Sa:So ratio in the kidneys. (**C**) Ratio of C22-24/C16 for ceramides in the kidneys. (**D**) Concentration in pmol/g of the kidney of 18:1/16:0. (**E**) Correlation of 18:1/16:0 with 18:2/16:0 in the kidneys. Results are expressed as a mean with standard error, n = 5 for Con and AC alone groups, and n = 6 for FB alone and AC + FB groups. ANOVA was used to estimate differences between groups. Statistically different groups are indicated by different letters (*p* < 0.05).

In the kidneys, FB1 was detectable only after 14 days of exposure in both FB- and FB + AC-fed pigs (Table 4). Given the variation in kidney weights between animals (Figure S1B), the Kidney:Feed ratio for FB1 was calculated. After excluding an extreme maximum value from each group (Grubbs test, *p* < 0.05), the average Kidney:Feed ratio for pigs fed with FB + AC was found to be 45% lower than that for pigs fed with FB, and this difference was statistically significant (Figure 3A).

The Sa:So ratio in the kidneys increased significantly by 59% in pigs fed with the FB diet for 14 days compared to those fed the control diet (Table 4). However, in pigs fed with FB + AC for 14 days, the Sa:So ratio did not significantly differ from controls. The observed increase of the Sa:So ratio in FB-fed pigs was primarily attributed to a 27% reduction of So levels, while the increase of Sa was smaller, at just 15% (Table S2).

The C22-24:C16 ratios for various SL classes in the kidney are presented in Table 4. Significant differences from controls were observed only in the pigs fed with FB for 14 days and in those fed with FB + AC for 9 days (Figure 3C). As seen in the liver, the effect of fumonisins on the C22-24:C16 ratio in the kidneys of pigs fed with FB for 14 days was linked to a significant decrease in 18:1/16:0 concentrations (Figure 3D). In contrast, in pigs fed with FB + AC for nine days, the increase in the C22-24:C16 ratio was not associated with a decrease in 18:1/16:0 (Figure 3D) but rather with an increase in C22-24 concentrations (Table S2).

The effects of fumonisins on 18:2/16:0 levels mirror those observed for 18:1/16:0 levels, although the changes were not statistically significant. Additionally, m18:1/16:0 and t18:0/16:0 were not detectable in the kidneys (Table S2). In the liver, a strong correlation between 18:1/16:0 and 18:2/16:0 was observed, with an R value of 0.85 (Figure 3E).

Regarding other sphingolipids in the kidney, the concentrations of most 18:1- and 18:2-ceramides were reduced after 14 days of exposure to the FB diet, with no clear pattern related to fatty acid chain length. In contrast, no significant difference in these analytes was observed in pigs fed the FB + AC diet for 14 days compared to controls. The effects of fumonisins on kidney concentrations of monohexosylceramides, lactosylceramides, and sphingomyelins were generally consistent with those seen in ceramides, although most of these changes were not statistically significant (Table S2).

### 2.5. Effect on Sphingolipids in the Lung

**Figure 4:**
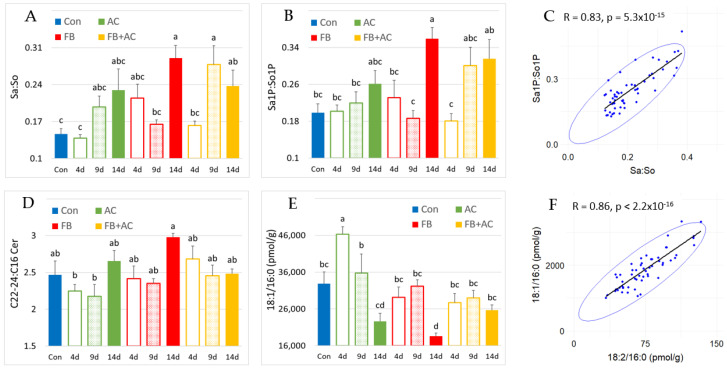
Effects in pig lungs of diets containing fumonisins (FB) and AlgoClay (AC) alone or in mixtures (AC + FB) for 4, 9, and 14 days on different biomarkers of fumonisin exposure and on C16-18:1 ceramide concentration. Con: control; Sa: sphinganine; So: sphingosine; Sa1P: sphinganine-1-phosphate; So1P: sphingosine-1-phosphate; Cer: ceramides. (**A**) Sa:So ratio in the lung. (**B**) Sa1P:So1P ratio in the lung. (**C**) Correlation between Sa1P:So1P and Sa:So ratios in the lung. (**D**) C22-24/C16 ratio for ceramides in the lung. (**E**) Concentration in pmol/g of the lung of 18:1/16:0. (**F**) Correlation of 18:1/16:0 with 18:2/16:0 in the lung. Results are expressed as a mean with standard error, n = 5 for Con and AC alone groups, and n = 6 for FB alone and AC + FB groups. ANOVA was used to estimate differences between groups. Statistically different groups are indicated by different letters (*p* < 0.05).

FB1 was not detected in the lungs of the pigs in this study. However, the Sa:So ratio was significantly increased by 98% in pigs that were fed the FB diet for 14 days, while the increase was 62% in pigs that were fed the FB + AC diet for the same duration (Figure 4A). These changes were primarily attributed to an increase in Sa concentrations (Table S3). The Sa1P:So1P ratio exhibited a similar pattern of variation to that of the Sa:So ratio (Figure 4B), and a strong correlation between these two biomarkers was observed in the lung, with an R value of 0.83 (Figure 4C).

The C22-24:C16 ratios measured for the different SL classes are presented in Table 5. A non-significant numerical increase in the C22-24:C16 ratio for ceramides was observed in the lungs of pigs fed the FB diet for 14 days (Figure 4D). This effect, similar to what was observed in the liver and kidneys, was associated with a significant decrease in 18:1/16:0 concentrations, which was more pronounced than the reduction observed for long-chain ceramides (Table S3, Figure 4E). In contrast, 18:1/16:0 concentrations in the lungs of pigs fed the FB + AC diet for 14 days were not significantly different from those in the control group (Figure 4E). A transient increase in the 18:1/16:0 concentration was noted at four days in pigs fed the AC diet, but this effect was not observed at 9 or 14 days. The effects of fumonisins on 18:1/18:0, 18:2/16:0, and 18:2/18:0 followed a similar pattern to 18:1/16:0, with significant reductions in concentrations observed in pigs fed the FB diet for 14 days. However, the concentrations in pigs fed the FB + AC diet for 14 days did not differ from those of the control group (Table S3). Consistent with the liver and kidney results, 18:1/16:0 concentrations were strongly correlated with 18:2/16:0 concentrations, with an R value of 0.86 between these two analytes (Figure 4F).

Regarding other sphingolipids, most ceramides and monohexosylceramides tended to decrease in pigs fed the FB diet for 14 days, whereas no such effect was observed in pigs fed the FB + AC diet for the same duration (Table S3).

### 2.6. Effect on Sphingolipids in the Plasma

**Figure 5:**
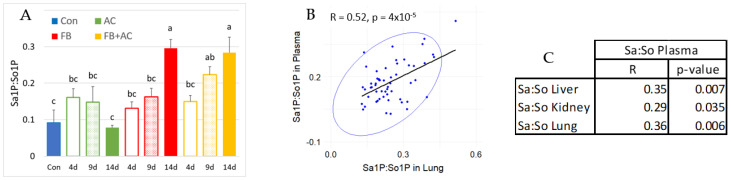
Effects in pig plasma of diets containing fumonisins (FB) and AlgoClay (AC) alone or in mixtures (AC + FB) for 4, 9, and 14 days on different biomarkers of fumonisins exposure. Con: control; Sa: sphinganine; So: sphingosine; Sa1P: sphinganine-1-phosphate; So1P: sphingosine-1-phosphate. (**A**) Plasma Sa1P:So1P ratio. (**B**) Correlation between Sa1P:So1P ratio in the plasma and lung. (**C**) Correlation between Sa:So ratio in plasma and Sa:So ratio in the liver, kidney, and lung. Results are expressed as a mean with standard error, n = 5 for Con and AC alone groups, and n = 6 for FB alone and AC + FB groups. ANOVA was used to estimate differences between groups. Statistically different groups are indicated by different letters (*p* < 0.05).

In this study, no significant differences were observed in the Sa:So ratio or plasma concentrations of Sa and So across the groups (Table S4). However, a significant increase in the Sa1P:So1P ratio was noted in pigs fed the FB diet for 14 days, as well as in pigs fed the FB + AC diet for both 9 and 14 days (Figure 5A).

The observed increase in the Sa1P:So1P ratio in plasma was primarily attributed to Sa1P, which showed a significant increase of 383% in pigs fed with FB and 349% in pigs fed with FB + AC for 14 days. As Sa1P and So1P were only quantifiable in the lung, a correlation analysis was performed between the Sa1P:So1P ratios measured in plasma and those in the lung. The correlation was found to be relatively strong, with an R value of 0.52 and a *p*-value of less than 0.001, indicating a significant relationship (Figure 5B). Further analysis revealed significant correlations between Sa:So ratios measured in plasma and those in the liver, kidney, and lung, although these correlations were weaker than those observed for the Sa1P:So1P ratios (Figure 5C).

No effects of fumonisins or AlgoClay, either alone or in combination, were found on the C22-24:C16 ratios measured for different sphingolipid classes in plasma. Similarly, no effect of fumonisins on plasma 18:1/16:0 concentrations was observed (Table S4). Interestingly, an increase in most ceramides and, to a lesser extent, sphingomyelins was noted in pigs fed the FB diet for nine days, a change not seen in pigs fed the FB + AC diet for the same duration (Table S4).

## 3. Discussion

### 3.1. Performance and FB1 in Tissues

At the conclusion of the study, a relative heterogeneity in animal weights was observed; however, no discernible effect on growth or feed consumption could be attributed to fumonisins or AlgoClay. Additionally, while differences were noted in certain biochemical variables at the plasma level between groups, these variations appeared to be transient and were not directly linked to any specific diet. These findings are in line with the low levels of fumonisins used in the diet [10], as well as with previous studies on chickens and pigs fed AlgoClay [20,39,40]. The observed changes in albumin and cholesterol concentrations in pigs fed the FB diet for nine days, as well as changes in creatinine, urea, and CPK concentrations in pigs fed the FB + AC diet for nine days, may indicate an effect unrelated to fumonisins or AlgoClay. The origin of this effect remains unclear, but it may help explain some of the discordant results observed in FB1 tissue concentrations and sphingolipid profiles in plasma after nine days of exposure, which will be discussed later.

In this study, a four-day administration of FB feed enabled the detection of FB1 in the liver, despite a fasting period of 14 to 16 h prior to slaughter. This is the first study to demonstrate the presence of FB1 in the liver after administering diets containing fumonisins at the maximum levels recommended for pigs in Europe (Table 6). The detection of FB1 in the liver thus serves as a highly sensitive biomarker for fumonisin exposure in this species. Given that direct comparisons of FB1 concentrations in tissues with studies in pigs are not available, comparisons were made with avian species, as their FB1 toxicokinetics are similar to those in pigs [18,19]. In chickens, the detection of FB1 in the liver was also reported as the most sensitive biomarker following the administration of a diet containing 7.5 mg FB1 + FB2/kg for 14 days [21]. Furthermore, in this study, the administration of the FB diet for 9 and 14 days resulted in a moderate increase in FB1 concentrations in the pig liver. Although this increase was not statistically significant, it aligns with data obtained from chickens, in which FB1 accumulation in the liver increased with prolonged exposure [20].

Additionally, FB1 was detected in the kidneys, but only in animals fed fumonisins for 14 days. The concentration of FB1 in the kidneys was approximately four times lower than in the liver, a finding consistent with observations in chickens, though differing from results obtained in rats [20,43,44]. Together with other mycotoxins, the measurement of FB1 in urine is commonly used in humans as a biomarker of exposure [2,3,4,5]. Unfortunately, for technical reasons, urine collection was not possible in this study. Compared with biomarkers measured in plasma or urine, the disadvantage of measuring mycotoxins in the liver or kidney is that the animals have to be slaughtered. However, this assay can easily be performed on samples collected at the slaughterhouse. In addition to providing an estimate of animal exposure and correlating the presence of mycotoxins in tissues with lesions or reduced performance, this analysis has the advantage of assessing the carry-over of mycotoxins and estimating the contribution of meat products to human exposure to mycotoxins [6,7,8,9].

### 3.2. Sa:So and Sa1P: So1P Ratios

**Figure 6:**
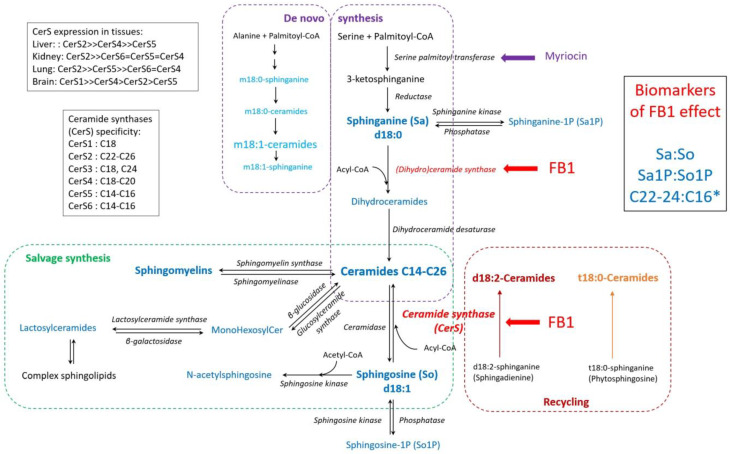
Simplified diagram of the three pathways responsible for the presence of sphingolipids in cells and the target of FB1 [22,46,47,48]. * C22-24:C16 ratios were measured on d18:0-, d18:1-, d18:2-, and m18:1-ceramides, d18:0- and d18:1-sphingomyelins, and on monohexosylcéramides and lactosylcéramides.

This study found no significant effect of fumonisins on the Sa:So ratio in plasma. The Sa:So ratio was the first biomarker characterized in animals to reveal fumonisin exposure [24]. Due to the direct inhibition of ceramide synthase (Cers) by FB1 (Figure 6), the increase in free sphinganine (Sa) is rapid [45]. In contrast, the increase in sphingosine (So) is variable and delayed, mainly due to the hydrolysis of sphingolipids from the plasma membrane (Figure 6), secondary to the decrease in cellular ceramides [22].

The effects of fumonisins on the Sa:So ratio are the most important biomarkers studied in pigs to date (Table 6). For instance, a rise in the Sa:So ratio was observed as early as six hours after a single oral administration of fumonisins at a dose of 2 mg/kg body weight [35]. Similarly, prolonged dietary exposure to fumonisins has shown an increase on the Sa:So ratio after nine days of consuming a diet containing 11.8 mg FB1 + FB2/kg [30] and after 14 days with a diet containing 7.2 mg FB1 + FB2/kg [34]. However, diets with fumonisin concentrations ranging from 2 to 4.4 mg only elicited a rise in the Sa:So ratio after 28 to 32 days of exposure [32,33,36]. Surprisingly, when diets containing 3.7, 8.1, and 12.2 mg FB1 + FB2/kg were fed for 28 days, an increase in the Sa:So ratio was observed only at the lowest concentration of 3.7 mg FB1 + FB2/kg [33]. These findings collectively suggest that low dietary concentrations of fumonisins can increase the Sa:So ratio in plasma, but only with prolonged exposure, and the increase does not necessarily correspond directly to the fumonisin concentration in the feed [33]. This observation aligns with data from studies conducted on ducks [49]. Interestingly, the co-administration of bentonite—a substance reported to bind fumonisins in some in vivo studies [50,51]—exacerbated the effects of fumonisins on the Sa:So ratio. The presence of bentonite increased the Sa:So ratio in the plasma, liver, kidney, and lung by 200–300% compared to fumonisin-only diets, although the effect was statistically significant only in the plasma and kidney [42].

For the first time in pigs, this study demonstrates that the plasma Sa1P:So1P ratio is a more sensitive biomarker than the Sa:So ratio. Sa1P and So1P are the phosphorylated forms of Sa and So following the action of a kinase (Figure 6). The demonstration of an increase in Sa1P:So1P is interesting because it corroborates previous studies in mice, humans, and chickens [25,26,27,28]. The strong correlations between plasma and lung Sa1P:So1P ratios suggest that the lung may be a major contributor to plasma Sa1P:So1P. Additionally, correlations between Sa:So ratios measured in plasma, liver, kidney, and lung suggest that these organs collectively influence variations in plasma Sa:So levels in pigs.

In the liver, the Sa:So ratio was significantly increased as early as nine days after exposure to fumonisins, whereas in the kidney, this increase was observed only after 14 days of exposure. These findings suggest that the liver is more sensitive to fumonisins’ effects on the Sa:So ratio than the kidney. This heightened sensitivity could be attributed to higher FB1 concentrations typically found in the liver compared to the kidneys. The hypothesis that tissue FB1 concentrations are responsible for organ sensitivity to fumonisins has previously been proposed in studies involving rats and chickens [43,44]. Interestingly, this study also observed an increase in pulmonary Sa:So after 14 days of exposure, despite the absence of detectable FB1 in the lung. Similar increases in pulmonary Sa:So have been reported in pigs administered different doses of fumonisins [23,31,42]. The finding that elevated pulmonary Sa:So occurs in the absence of detectable FB1 aligns with earlier research in chickens [43]. This phenomenon has led to the hypothesis of an indirect effect of fumonisins or their action on specific cell types. In pigs, fumonisins have been suggested to target specialized endothelial cells, explaining the distinctive histological lesions observed [52]. Overall, these results highlight the complexity of fumonisins’ effects on the Sa:So ratio, which appear to be strongly influenced by the duration of exposure rather than directly linked to FB1 concentrations in tissues.

### 3.3. Ceramides and Other Sphingolipids

The administration of a fumonisin-containing diet resulted in a decrease in total ceramide concentrations in the liver, kidney, and lung after 14 days. However, a transient increase in ceramide levels was observed in the plasma of pigs fed the FB diet for nine days. This temporary increase in plasma ceramides coincided with elevated cholesterol and protein concentrations and was attributed to an unexpected effect specific to this time point. In contrast, the observed reduction in ceramide levels in tissues aligns with extensive previous research and is consistent with the known inhibitory effect of fumonisins on ceramide synthases [22]. Interestingly, the FB diet predominantly reduced C16 ceramides, while its effects on C22-24 ceramides were variable. The decrease in C16 ceramides was evident across the liver, kidney, and lung for 18:1-ceramides, as well as for 18-2- and m18-1-ceramides. However, 18:0- and t18:0-ceramides were unaffected by fumonisins. These findings are consistent with earlier studies on chickens [25] and highlight the differential sensitivity of ceramides to fumonisins, which appears to depend on the sphingoid base (Figure 6).

It is noteworthy that variable effects were observed for C22-24 ceramides, with a slight increase detected in the liver of pigs fed FB diets for 9 days, followed by a decrease at 14 days. A similar transient, compensatory increase in C22-24 ceramides has been reported in the livers of chickens exposed to fumonisins for a short duration, but this effect dissipated with prolonged exposure [25,28,29,43,53,54]. To date, only one previous study has explored the effects of fumonisins on the sphingolipidome in pigs [31]. In this earlier study, pigs were administered a daily gavage of fumonisins at a dose equivalent to a diet containing 20–25 mg FB1 + FB2/kg for nine days. The results showed a reduction in the relative abundance, though not the absolute concentrations, of C16 ceramides in the liver. In contrast, C22-24 ceramide concentrations were reduced in the lungs [31]. The discrepancies between these studies may be attributed to differences in fumonisin dosages. Collectively, these findings underscore the differential sensitivity of ceramides to fumonisins, which appears to depend on the fatty acid composition.

This observation is important because the cytotoxicity of ceramides varies greatly [55,56,57]. Dihydroceramides and ceramides are formed by the acylation of sphinganine and sphingosine with a fatty acid (Figure 6). Deoxyceramides are derived from deoxysphinganine (m18:0), which is synthesised in cells by the incorporation of alanine in place of serine, following the same pattern as ceramides derived from sphinganine and sphingosine (Figure 6) [58]. Sphingadienine (d18:2), derived from the diet or obtained by reducing sphingosine, leads to the formation of d18:2-ceramides [59]. Phytosphingosine (t18:0), found in yeast, is also synthesised by mammals and leads to the formation of t18:0-ceramides [60]. Several ceramide synthases (CerS) have been characterized that differ in chain length specificity and tissue expression (Figure 6). CerS2 is responsible for the formation of C22-24 ceramides, whereas CerS5 is responsible for the formation of C16 ceramides [48,61].

In this study, the reduction in C16 ceramide concentrations resulted in an increase in the C22-24:C16 ratio in the liver and kidney after 14 days of FB feed administration. This increase was approximately 33% in the liver but only 19% in the kidney, where FB1 concentrations were four times lower. Several previous studies have proposed using the C22-24:C16 ratio as a biomarker for fumonisin effects on chickens, alongside Sa:So and Sa1P:So1P ratios [25,29]. One notable advantage of this biomarker is its potential to rely more directly on tissue FB1 concentrations than Sa:So, which primarily reflects the duration of fumonisin exposure in chickens [29,49,62]. The findings from this study on pigs appear to support and extend these observations on chickens [43], suggesting that the C22–C24:C16 ratio may serve as a valuable biomarker for assessing fumonisin effects on the liver and kidney but not on the lung.

The relative abundance in ceramides is considered a key element in the cell homeostasis and in the toxicity of fumonisins [22]. A decrease in C22-24 ceramides in genetically CerS2-deficient mice has been reported to induce signs of hepatotoxicity similar to those observed with FB1 [63,64]. In pigs receiving 5 mg FB1/kg BW daily for 9 days, variable CerS expression and activity were suggested to explain the greater susceptibility of the lung compared to the liver [31]. In a porcine jejunal epithelial cell line, 40 and 80 µM FB1 specifically inhibited Cers2 expression, leading to the production of pro-inflammatory cytokines and endoplasmic reticulum stress [65]. Together with the hypothesis of an indirect effect of fumonisins or their action on specific cell types, the different effects of fumonisins on ceramides help to explain why, despite a very sharp increase in Sa concentrations, the liver and kidney are less sensitive to FB1 toxicity than the lung, which is proportionally less affected by the increase in Sa concentrations [24]. Results in pigs are consistent with those observed in chickens, where low tissue FB1 concentrations lead to a decrease in C16 ceramides only, whereas higher levels lead to a concomitant decrease in C16 and C22-24 ceramides [29].

### 3.4. Effects of AlgoClay Distribution

The FB + AC diet led to a 43% and 29% reduction in the Liver:Feed ratio of FB1 at four and 14 days of exposure, respectively, compared to the FB diet. Although the reduction in the liver was not statistically significant, the observed trends are in line with previous studies on chickens, which reported a 40% decrease in liver FB1 concentrations after nine days of exposure [20]. The absence of a significant effect on the Liver:Feed ratio at nine days in this study may be due to confounding factors, which could also explain the observed increases in creatinine, urea, and CPK levels in plasma at the same time point. For the kidney, the FB + AC diet resulted in a 45% reduction in the Kidney:Feed ratio of FB1 at 14 days compared to the FB diet. This result is close to the overall reduction in urinary FB1 of 45% at 12 h and 55% at 24 h observed in rats when 0.25% montmorillonite clay was added to the diet [50]. Urinary excretion was also reduced in humans given the clay compared to the placebo [50].

In contrast, the FB + AC diet did not affect the Sa:So or Sa1P:So1P ratios in any tissue, compared to the FB diet. This finding is consistent with earlier studies on chickens [20], as well as studies on pigs and poultry, which indicated that fumonisin-induced alterations in Sa:So and Sa1P:So1P ratios do not directly reflect the FB1 tissue concentrations. In line with this, the administration of bentonite alongside fumonisins has been shown to increase Sa:So levels in plasma and tissues, with this effect being more pronounced than in feeds containing fumonisins alone, despite bentonite’s known ability to bind fumonisins [42,50,51].

In this study, feeding the FB + AC diet generally prevented the decrease in C16 ceramide concentrations seen with the FB diet at 14 days. This effect was observed for 18:1-, 18:2-, and m18:1-ceramides in the liver, kidney, and lung. Interestingly, a slight increase in C22-24 ceramide concentrations was observed in the liver of pigs fed the FB + AC diet at 14 days. This contrasts with the FB diet, in which a slight increase in C22-24 concentrations at nine days was followed by a decrease at 14 days. These findings are noteworthy because a decrease in C22-24 ceramide production has been considered a key factor in fumonisin toxicity [22,31,65]. More generally, and independent of fumonisin intake, the relative abundance of long and very long chain ceramides and sphingomyelin is now considered a key issue in disease and toxicity in humans [66,67,68].

All together, these results align with previous findings in poultry, suggesting that the effects of fumonisins on ceramides are influenced by the sphingoid base, the fatty acid, and the FB1 concentration in tissues [29]. Consequently, the C22-24:C16 ratio for 18:1-, 18:2-, and m18:1-ceramides would primarily reflect tissue FB1 concentrations, while 18:0- and t18:0-ceramides appear to be less sensitive. In contrast, the Sa:So and Sa1P:So1P ratios seem more directly influenced by the duration of exposure to low doses of fumonisins in feed. Further studies are needed to validate these observations and explore the underlying mechanisms.

## 4. Conclusions

In conclusion, this study demonstrates that FB1, detectable in the liver after just four days of exposure, serves as the most sensitive biomarker for pigs fed a diet containing fumonisins at the maximum dose recommended in Europe. In plasma, the Sa1P:So1P ratio emerges as the most sensitive biomarker, though it only shows a significant increase after 14 days of exposure. The majority of Sa:So and C22-24:C16 ratios, measured for 18:1-, 18:2-, and m18:1-ceramides in the liver, kidney, and lung, were elevated only after 14 days, with the fumonisin-induced changes in C22-24:C16 ratios linked to a reduction in C16 ceramides. While the effects of fumonisins on sphingolipids in the lung mirrored those observed in the liver, FB1 was undetectable in the lung tissue. When comparing diets containing fumonisins alone to those supplemented with AlgoClay, the presence of AlgoClay slightly modified the effects of fumonisins on Sa:So and Sa1P:So1P ratios, despite that the tissue-to-feed ratios for FB1 were reduced, and the decrease in C16 ceramides was no longer evident. Overall, these findings suggest that the effects of fumonisins on sphingolipids in the liver, kidney, and lung are not solely dependent on tissue FB1 concentrations, and the Sa:So ratio alone does not fully capture the complex effects of fumonisins in pigs. Further studies are necessary to understand the mechanisms of the effects of AlgoClay on the sphingolipidome.

## 5. Materials and Methods

### 5.1. Feed Preparation

The experimental diets were designed to meet the physiological needs of the animals. Four diets were formulated by Research Diet Services (Research Diet Services, Wijk bij Duurstede, Netherland): a mycotoxin-free control diet (Con), a diet containing fumonisins (FB) at an expected concentration of 5 mg FB1 + FB2/kg, a diet containing fumonisins at an expected concentration of 5 mg FB1 + FB2/kg combined with 450 mg/kg AlgoClay (FB + AC), and a diet containing 450 mg/kg AlgoClay alone (AC). The FB and FB + AC diets were created by incorporating 2.5% maize meal naturally contaminated with 200 mg FB1 + FB2/kg, in accordance with EU regulations (2006/576/EC). Mycotoxins in the diets were quantified by LC-MS/MS based on the AFNOR standard V03-110 [69], carried out by Labocéa (Labocéa, Ploufragan, France). The measured concentrations of fumonisins in the diet were 5.29 and 5.75 mg FB1 + FB2/kg for the FB and the FB + AC diets, respectively. Except for fumonisins, all other mycotoxins analyzed were either undetectable or present at trace levels (Table S5).

### 5.2. Animal Phase

The animal phase was conducted at Cebiphar (Cebiphar, Fondettes, France) in accordance with the European Directive EC2010/63 on the care and use of animals in research, under project number #25251-202004301110247, and it was approved by the French Ministry of Higher Education, Research and Innovation on 3 November 2020. Fifty-six, 21-day-old male Large White x Landrace pigs, supplied by SAS Pottier (SAS Pottier, Fléré-La-Rivière, France), were acclimatized for one week on a mycotoxin-free commercial weaning diet. The animals were weighed and allocated into 10 homogeneous groups of five or six pigs housed in 10 indoor pens. All pigs not receiving the FB, FB + AC, or AC diets were fed the control diet, along with five pigs from day 1 to day 14. The FB and FB + AC diets were fed to 36 animals, divided into three groups of six pigs per diet for four, nine, and 14 days, while the AC diet was fed to 15 animals, divided into three groups of five pigs for the same durations. The animals were slaughtered on three consecutive days, as detailed in Table S6. Drinking water was provided ad libitum, with one trough per pen. Feed was also provided ad libitum, with one feeder per pen throughout the study. All pens were maintained under similar environmental conditions. Feed consumption was recorded daily for each group, and animals were weighed on days 1, 7, and 13. Clinical observations were conducted once daily. A fasting period from 14:00 to 16:00 was observed before blood sampling and intravenous sacrifice using T61 (MSD).

### 5.3. Sample Collection and Blinding

Blood samples were collected on the day of sacrifice using sterile, disposable 22G needles in lithium heparinized tubes, following CEBIPHAR SOPs. The collected blood was centrifuged at 2500 rpm for 10 min at approximately +5 °C in a refrigerated centrifuge. Plasma samples were stored at −20 °C for up to 2 h after collection. Immediately following sacrifice, the left lobe of the liver, the left kidney, and the median lobe of the left lung were removed, examined macroscopically, and then stored at −80 °C. No lesions or abnormalities were observed during the examination.

The diet distribution was not performed blindly at the test facility; however, all sample analyses were conducted blindly. The analyst knew the animal group assignments but was unaware of the corresponding diets assigned to the groups. The anonymity of the animal groups was maintained until all samples had been analyzed.

### 5.4. Chemicals and Reagents

The analytes and reagents used in this study were sourced from Sharlab (Sharlab S.L., Sentmenat, Spain) or Sigma (Sigma-Aldrich Chimie SARL, Saint Quentin Fallavier, France). All reagents and solvents were of HPLC grade, except for those used in the determination of mycotoxins and sphingolipids, which were of LC-MS grade. Standards for mycotoxins in food were purchased from Sigma or Biopure (Romer Labs, Getzersdorf, Austria). Certified solutions of [13C34]-FB1, [13C34]-FB2, and [13C34]-FB3 used as internal standards (IS) for fumonisin determination in liver, kidney, and lung were obtained from Biopure™. FUMONIPREP^®^ columns were supplied by R-Biopharm (R-Biopharm Rhone LTD, Glasgow, Scotland). Sphingolipid standards were acquired from Sigma. The IS mixture used was Avanti Polar Lipids’ ‘Ceramide/Sphingoid Internal Standard Mixture I’, which included C17-sphingosine, C17-sphinganine, C17-sphingosine-1 P, C17-sphinganine-1 P, C12:0-lactosyl(ß)-ceramide, C12:0-sphingomyelin, C12:0-glucosyl(ß)-ceramide, C12:0-ceramide, C12:0-ceramide-1 P, and C25:0-ceramide, supplemented by m17:1/12:0, m18:1/12:0, and C12:0-ceramide sulfatide.

### 5.5. Biochemistry

Plasma samples were analyzed using a KONELAB 20 clinical chemistry analyzer (Fisher Scientific SAS, Illkirch, France), following the manufacturer’s instructions. The following biochemical parameters were measured: uric acid, cholesterol, total protein, albumin, globulin, lactate dehydrogenase (LDH, EC 1.1.1.27), alkaline phosphatase (ALP, EC 3.1.3.1), alanine aminotransferase (ALT, EC 2.6.1.2), aspartate aminotransferase (AST, EC 2.6.1.1), and creatinine phosphokinase (CPK, EC 2.7.3.2).

### 5.6. Tissue Concentrations of Fumonisins

FB1, FB2, and FB3 concentrations in liver, kidney, and lung tissues were determined by UHPLC-MSMS analysis, as previously described [43,70]. Briefly, one gram of tissue was homogenized in 2 mL of 0.9% NaCl using an Ultra-Turrax^®^ device. To this, 2 mL of acetonitrile/methanol (1:1) and 20 µL of an IS mixture were added. The samples were placed on a shaking table for 2 h, followed by centrifugation at 3000× *g* for 15 min to obtain the supernatant. After lipid extraction with hexane, the aqueous phase was subjected to FUMONIPREP^®^ column extraction, according to the manufacturer’s instructions. The eluent was collected and evaporated to dryness. UHPLC conditions, multiple reaction monitoring (MRM) parameters, retention times, and method validation are detailed in [70]. Fumonisin concentrations in the lungs were below 0.25 ng FB1/g, and FB2 and FB3 concentrations were below 1 ng/g in all tissues, which are the LOD of the method. FB1 concentrations were quantified by quadratic regression against standard curves, using Agilent MassHunter Quantitative Analysis software B.05.291.0. Liver:Feed and Kidney:Feed ratios in FB1 were calculated for each animal to account for differences in fumonisin concentrations between the FB and FB + AC diets, as well as variations in liver and kidney weights (Figure 1A).

### 5.7. UHPLC-MSMS for Sphingolipids

Sphingolipid extraction from liver, kidney, lung, and plasma was performed as previously described [28,43]. Briefly, 1 g of tissue was homogenized in 3 mL of phosphate buffer (0.1 M, pH 7.4) using a Potter-Elvehjem homogenizer, followed by centrifugation at 3000× *g* for 15 min. A 40 µL aliquot of the supernatant was diluted with 120 µL of 0.9% NaCl and spiked with 10 µL of an IS solution to a final concentration equal to 6250 pmol/g of tissue. To this, 600 µL methanol/chloroform (2/1, *v*/*v*) was added. After overnight incubation at 48 °C, 100 µL of 1 M KOH in methanol was added to cleave potentially interfering glycerolipids [71], and the samples were incubated for 2 h at 37 °C. Subsequently, 10 µL of 50% acetic acid was added. The samples were then centrifuged, the supernatant collected, and the residue was extracted again. The two supernatants were pooled and evaporated to dryness, and the dry residue was dissolved in 200 µL of methanol. After filtration, a 5 µL aliquot was injected into the chromatography system. Plasma sphingolipids were extracted using the same method with 60 µL of plasma and 100 µL of 0.9% NaCl.

Analyte separation was achieved on a Poroshell 120 column from Agilent (3.0 × 50 mm, 2.7 µm) using an Agilent 1260 autosampler binary pump (Agilent, Santa Clara, CA, USA), at a flow rate of 0.3 mL/min as described previously [28]. Detection was performed by dynamic MRM using an Agilent 6410 triple quadrupole spectrometer, with positive electrospray ionization at 300 °C, at a gas flow rate of 10 L/min, and a capillary voltage of 4000 V under 25 psi. The analytes analyzed correspond to those described in [25].

Chromatograms were analyzed using Agilent MassHunter quantitative analysis software B.05.291.0, with quadratic regression and a weighting factor of 1/x^2^ according to [72]. The method demonstrated good linearity across a wide concentration range (Table S8), consistent with previous findings [25]. Precision was deemed acceptable, with a relative standard deviation (RSD) of 20%. Intra-day repeatability, assessed by the RSD of recovery measured on the IS, is detailed in Table S7. A value of less than 20% was considered acceptable. For sphingolipids lacking available standards, concentrations were calculated using calibration curves derived from analytes within the same class and of similar mass. Final concentrations in the liver, kidney, and plasma were adjusted for recovery as measured using the corresponding IS (Table S8).

### 5.8. Statistics

Data are presented as mean ± SD in the Tables, while Figures display error bars as SE (SD/square root of the sample size) for improved clarity. All statistical analyses were conducted using R 4.3.0 (www.r-project.org). Two grouping strategies were used to analyze differences between groups. In the first analysis strategy, between-group comparisons were made on the 10 experimental groups. In a second strategy, pigs not exposed to fumonisins, corresponding to groups Con, AC 4 d, AC 9 d, and AC 14 d, were grouped together to be compared with the 6 groups receiving diets contaminated with fumonisins. As the interpretation of the results did not differ between the two analysis strategies, the first strategy was presented in the manuscript, while the results of the statistical analysis performed with the second strategy are presented in Table S9.

The Shapiro test for normality was first performed on the entire data set. If the data followed a normal distribution, group differences were assessed using ANOVA. If the data did not follow a normal distribution, a logarithmic transformation was performed, and the normality test was performed. If the logarithmically transformed data followed a normal distribution, group differences were assessed by ANOVA. If the log-transformed data did not follow a normal distribution, a Grubbs test was performed to exclude outlying data and the analysis procedure described above was repeated. If the data without outliers followed a normal distribution, group differences were assessed using ANOVA. If the logarithmically transformed data without outliers did not follow a normal distribution, a Kruskal–Wallis test was performed. After each ANOVA, a post-hoc test such as Student–Newman–Keuls was performed to identify statistically different groups. A *p*-value < 0.05 was considered statistically significant.

## Figures and Tables

**Table 1:** Effects of feeding diets containing fumonisins (FB) and AlgoClay (AC) alone or in mixtures (FB + AC) for 4, 9, and 14 days on the performance of pigs.

Performance	Con	AC 4 d	AC 9 d	AC 14 d	FB 4 d	FB 9 d	FB 14 d	FB + AC 4 d	FB + AC 9 d	FB + AC 14 d
BW D1 (kg)	7.06 ± 1.21	7.04 ± 1.32	7.28 ± 1.69	6.95 ± 1.4	7.22 ± 1.59	7.06 ± 1.18	6.79 ± 0.76	7.32 ± 1.02	7.24 ± 1.52	7.41 ± 0.93
BW D13 (kg)	11.2 ± 2.0	10.7 ± 2.6	11.1 ± 3.7	10.3 ± 2.0	11.4 ± 3.3	11.1 ± 2.4	11.4 ± 1.0	11.2 ± 1.7	10.4 ± 2.6	10.9 ± 2.7
FI (kg)	33.8	33.8	34.6	35.1	40.8	43.1	39	38.6	43.2	38
Liver (g)	424 ± 98	488 ± 134	479 ± 133	424 ± 144	433 ± 149	499 ± 128	458 ± 25	404 ± 56	432 ± 122	399 ± 123
Kidney (g)	61.1 ± 10.3	71.9 ± 10.9	77.1 ± 23.1	66.8 ± 14.9	70.7 ± 15.5	70.2 ± 9.2	65 ± 11.4	66.6 ± 11.6	62 ± 16.9	63.5 ± 12.8
Liver (%)	3.76 ± 0.34 ^ab^	4.56 ± 0.35 ^a^	4.38 ± 0.51 ^ab^	4.06 ± 0.76 ^ab^	3.76 ± 0.58 ^ab^	4.5 ± 0.35 ^ab^	4.04 ± 0.37 ^ab^	3.62 ± 0.1 ^b^	4.12 ± 0.32 ^ab^	3.64 ± 0.5 ^b^
Kidney (%)	0.55 ± 0.06	0.69 ± 0.09	0.7 ± 0.08	0.65 ± 0.11	0.64 ± 0.1	0.65 ± 0.11	0.57 ± 0.08	0.58 ± 0.03	0.59 ± 0.04	0.59 ± 0.05

BW: body weight; FI: feed intake from day 1 to day 14; D: day of experiment. Results are expressed as mean ± SD, n = 5 for Con and AC groups, and n = 6 for FB and FB + AC groups. ANOVA was used to estimate differences between groups. Different letters indicate statistically different groups (*p* < 0.05).

**Table 2:** Effects of feeding diets containing fumonisins (FB) and AlgoClay (AC) alone or in mixtures (FB + AC) for 4, 9, and 14 days on blood biochemistry in pigs.

Groups	Con	AC 4d	AC 9 d	AC 14 d	FB 4 d	FB 9 d	FB 14 d	FB + AC 4 d	FB + AC 9 d	FB + AC 14 d
Total protein (g/L)	57.5 ± 7.5 ^abc^	61.4 ± 3.3 ^abc^	53.6 ± 14.5 ^bc^	49.6 ± 5.4 ^c^	53.8 ± 4.3 ^bc^	69.4 ± 5.7 ^a^	57.9 ± 1.5 ^bc^	59.2 ± 4.2 ^abc^	63.5 ± 5.4 ^ab^	61.9 ± 17.1 ^ab^
Albumin (g/L)	34.8 ± 2.2 ^b^	36.8 ± 3.1 ^ab^	35.1 ± 2.2 ^b^	33.1 ± 3.9 ^b^	33.4 ± 4.2 ^b^	42.1 ± 3 ^a^	35.8 ± 1.2 ^b^	31.8 ± 6.2 ^b^	32.9 ± 11.4 ^b^	33.5 ± 3.9 ^b^
Cholesterol (mmol/L)	2.82 ± 0.59 ^bc^	2.88 ± 0.4 ^bc^	3.17 ± 0.56 ^b^	2.41 ± 0.34 ^c^	2.82 ± 0.49 ^bc^	3.83 ± 0.8 ^a^	2.9 ± 0.46 ^bc^	3.06 ± 0.32 ^bc^	2.87 ± 0.42 ^bc^	2.88 ± 0.37 ^bc^
Triglycerides (mmol/L)	0.81 ± 0.24	0.94 ± 0.31	0.66 ± 0.46	0.67 ± 0.08	0.57 ± 0.2	1.26 ± 0.4	1.12 ± 0.55	0.7 ± 0.11	1.11 ± 0.63	0.65 ± 0.37
Urea (mmol/L)	2.92 ± 1 ^b^	2.43 ± 1.05 ^b^	3.82 ± 2.5 ^ab^	2.85 ± 1.34 ^b^	2.74 ± 1.04 ^b^	3.26 ± 1.57 ^b^	2.58 ± 0.86 ^b^	2.77 ± 0.97 ^b^	5.06 ± 2.25 ^a^	4.09 ± 0.75 ^ab^
Creatinine (µmol/L)	81.7 ± 14 ^b^	73.2 ± 10.5 ^b^	87.9 ± 20.8 ^ab^	81.9 ± 43.1 ^b^	73 ± 16 ^b^	86.2 ± 7.4 ^ab^	68.9 ± 8.8 ^b^	77.1 ± 12.6 ^b^	109 ± 30 ^a^	82.5 ± 17.1 ^b^
LDH (UI)	1394 ± 224 ^ab^	1359 ± 226 ^ab^	1145 ± 348 ^b^	1278 ± 180 ^ab^	1293 ± 216 ^ab^	1632 ± 332 ^a^	1423 ± 316 ^ab^	1334 ± 349 ^ab^	1573 ± 326 ^a^	1443 ± 157 ^ab^
ASAT (UI)	42.8 ± 9.4	51.2 ± 8.6	42.1 ± 4.8	42.2 ± 8.1	46 ± 5.9	52 ± 5.3	56.2 ± 23.1	50.6 ± 2.2	57.4 ± 17.3	46 ± 13.6
ALAT (UI)	32.7 ± 2.1 ^abc^	35.1 ± 8.9 ^abc^	31.2 ± 6.3 ^bc^	32.1 ± 6 ^abc^	34.7 ± 7.1 ^abc^	37.9 ± 7.8 ^ab^	36.7 ± 5.2 ^ab^	28.8 ± 3.2 ^c^	39.7 ± 5.9 ^a^	31.3 ± 3.8 ^bc^
ALP (UI)	868 ± 194 ^ab^	1067 ± 207 ^a^	709 ± 262 ^bc^	682 ± 182 ^bc^	719 ± 175 ^bc^	823 ± 215 ^ab^	638 ± 201 ^bc^	637 ± 111 ^bc^	821 ± 217 ^ab^	562 ± 162 ^c^
CPK (UI)	590 ± 482 ^c^	1695 ± 1135 ^a^	471 ± 203 ^c^	622 ± 344 ^bc^	460 ± 36 ^c^	712 ± 265 ^bc^	829 ± 633 ^bc^	576 ± 116 ^bc^	1306 ± 1091 ^ab^	442 ± 128 ^c^

LDH = lactate dehydrogenase; ASAT = aspartate aminotransferase; ALAT = alanine aminotransferase; ALP = alkaline phosphatase; CPK = creatinine phosphokinase. Results are expressed as mean ± SD, n = 5 for Con and AC groups, and n = 6 for FB and FB + AC groups. ANOVA was used to assess differences between groups. Statistically different groups are indicated by different letters (*p* < 0.05).

**Table 3:** Effects of feeding diets containing fumonisins (FB) and AlgoClay (AC) alone or in mixtures (FB + AC) for 4, 9, and 14 days on the FB1 concentration, Sa:So ratio, C16, Sum of C22-24, Sum, and C22-24:C16 ratio from different classes of sphingolipids on the liver of pigs.

Analytes	Con	AC 4 d	AC 9 d	AC 14 d	FB 4 d	FB 9 d	FB 14 d	FB + AC 4 d	FB + AC 9 d	FB + AC 14 d	*p*-Value
FB1 (ng/g of liver)	ND	ND	ND	ND	1.90 ± 0.93	2.18 ± 1.41	2.65 ± 1.05	1.37 ± 0.60	2.72 ± 0.93	2.24 ± 1.36	0.140
Sa:So	0.04 ± 0.01 ^c^	0.05 ± 0.01 ^c^	0.04 ± 0.01 ^c^	0.06 ± 0.02 ^bc^	0.05 ± 0.01 ^c^	0.09 ± 0.02 ^ab^	0.12 ± 0.01 ^a^	0.06 ± 0.01 ^bc^	0.09 ± 0.03 ^ab^	0.14 ± 0.06 ^a^	<0.001
18:1/16:0	68.9 ± 12.2 ^a^	73.9 ± 20.4 ^a^	79.5 ± 6.6 ^a^	67.6 ± 13.4 ^a^	74.1 ± 10.4 ^a^	86.6 ± 11.5 ^a^	47.2 ± 11.2 ^b^	80.5 ± 19 ^a^	85.1 ± 19.1 ^a^	71.8 ± 27.7 ^a^	0.001
18:2/16:0	3.07 ± 0.66 ^a^	4.07 ± 1.25 ^a^	4.55 ± 0.57 ^a^	3.34 ± 0.62 ^a^	3.66 ± 0.9 ^a^	4.3 ± 1.22 ^a^	2.11 ± 0.24 ^b^	3.96 ± 0.85 ^a^	4.29 ± 1.12 ^a^	3.63 ± 1.88 ^a^	0.001
m18:1/16:0	8.4 ± 3.5	8.15 ± 2.49	8.32 ± 1.49	7.26 ± 2.16	8.09 ± 1.62	9.21 ± 3	4.41 ± 1.53	8.68 ± 2.72	8.9 ± 1.02	7.97 ± 3.3	0.117
Hex18:1/16:0	7.96 ± 0.8 ^ab^	6.6 ± 1.27 ^ab^	7.97 ± 2.55 ^ab^	7.06 ± 1.06 ^ab^	7.53 ± 1.85 ^ab^	5.34 ± 1.44 ^ab^	4.96 ± 0.99 ^b^	8.72 ± 1.89 ^a^	6.61 ± 1.41 ^ab^	6.53 ± 2.92 ^ab^	0.010
SM18:1/16:0	55.5 ± 4.7	50.9 ± 11.3	54.6 ± 13.6	58.5 ± 16.8	60.8 ± 10.2	45.8 ± 13.4	49 ± 11.5	62.6 ± 16.4	51 ± 14.5	60.5 ± 25.4	0.618
Sum C22-24 Cer	139 ± 21 ^ab^	148 ± 37 ^ab^	149 ± 17 ^ab^	147 ± 23 ^ab^	159 ± 14 ^ab^	176 ± 24 ^ab^	125 ± 21 ^b^	172 ± 26 ^ab^	183 ± 29 ^a^	173 ± 61 ^ab^	0.020
Sum C22-24 d18:2-Cer	9.4 ± 1.3 ^ab^	10 ± 2.6 ^ab^	11.6 ± 2 ^a^	8.7 ± 2.8 ^ab^	11.4 ± 2.9 ^a^	10.8 ± 3.1 ^a^	6.4 ± 1 ^b^	10 ± 1.5 ^ab^	10.8 ± 2.1 ^a^	9.7 ± 4.4 ^ab^	0.015
Sum C22-24 m18:1-Cer	0.6 ± 0.1	0.6 ± 0.2	0.7 ± 0.1	0.8 ± 0.1	0.7 ± 0.1	0.9 ± 0.3	0.6 ± 0.2	0.7 ± 0.1	0.9 ± 0.1	0.8 ± 0.3	0.039
Sum C22-24 HexCer	24.9 ± 2.2	21.3 ± 4.1	24.3 ± 5.3	21.2 ± 3.5	24.6 ± 5.3	19.6 ± 3.9	20.5 ± 3.2	25.7 ± 4.2	23.3 ± 3.5	25.8 ± 9.5	0.336
Sum C22-24 SM	237 ± 45	232 ± 76	220 ± 83	242 ± 80	278 ± 61	219 ± 67	256 ± 77	291 ± 90	252 ± 95	294 ± 140	0.659
Sum Cer	277 ± 45 ^ab^	288 ± 70 ^ab^	295 ± 25 ^ab^	284 ± 42 ^ab^	309 ± 30 ^ab^	345 ± 42 ^a^	233 ± 40 ^b^	329 ± 53 ^a^	351 ± 57 ^a^	316 ± 106 ^ab^	0.010
Sum d18:2-Cer	14.4 ± 2.2 ^ab^	16.4 ± 4.4 ^a^	19.1 ± 2.3 ^a^	13.8 ± 3.8 ^ab^	17.6 ± 4.2 ^a^	18.2 ± 5.3 ^a^	9.9 ± 1.3 ^b^	16.7 ± 2.6 ^a^	18.3 ± 3.7 ^a^	16.1 ± 7.6 ^ab^	0.004
Sum m18:1-Cer	9.01 ± 3.55	8.78 ± 2.61	9.04 ± 1.6	8.04 ± 2.2	8.77 ± 1.66	10.1 ± 3.1	4.99 ± 1.68	9.42 ± 2.78	9.77 ± 1.07	8.76 ± 3.55	0.117
Sum HexCer	35.1 ± 3.6	29.8 ± 5.9	34.1 ± 7.7	30 ± 4.8	34 ± 7.5	26.6 ± 5.4	27.4 ± 4.4	36.9 ± 6.9	32 ± 4.9	34.1 ± 12.3	0.197
Sum SM	397 ± 67	377 ± 115	373 ± 129	406 ± 126	461 ± 95	362 ± 108	411 ± 110	483 ± 144	418 ± 146	484 ± 227	0.630
Ratio C22-24:C16 Cer	2.03 ± 0.18 ^bc^	2.03 ± 0.15 ^bc^	1.88 ± 0.2 ^c^	2.21 ± 0.22 ^bc^	2.18 ± 0.27 ^bc^	2.04 ± 0.15 ^bc^	2.69 ± 0.3 ^a^	2.17 ± 0.2 ^bc^	2.18 ± 0.2 ^bc^	2.43 ± 0.29 ^ab^	<0.001
Ratio C22-24:C16 d18:2-Cer	3.1 ± 0.37	2.5 ± 0.24	2.59 ± 0.57	2.58 ± 0.57	3.15 ± 0.4	2.52 ± 0.21	3.04 ± 0.38	2.57 ± 0.38	2.56 ± 0.27	2.76 ± 0.5	0.053
Ratio C22-24:C16 m18:1-Cer	0.08 ± 0.04 ^b^	0.08 ± 0.02 ^b^	0.09 ± 0.01 ^ab^	0.11 ± 0.04 ^ab^	0.09 ± 0.02 ^ab^	0.1 ± 0.03 ^ab^	0.14 ± 0.02 ^a^	0.09 ± 0.03 ^ab^	0.1 ± 0.02 ^ab^	0.1 ± 0.02 ^ab^	0.024
Ratio C22-24:C16 HexCer	3.14 ± 0.15 ^cd^	3.23 ± 0.25 ^bcd^	3.13 ± 0.44 ^cd^	3.01 ± 0.26 ^d^	3.31 ± 0.28 ^bcd^	3.72 ± 0.36 ^ab^	4.17 ± 0.38 ^a^	2.98 ± 0.21 ^d^	3.6 ± 0.62 ^abc^	4.22 ± 1.36 ^a^	0.001
Ratio C22-24:C16 SM	4.18 ± 0.57	4.39 ± 0.73	3.86 ± 0.71	4.02 ± 0.55	4.47 ± 0.49	4.67 ± 0.7	5.09 ± 0.71	4.52 ± 0.46	4.78 ± 0.72	4.76 ± 0.95	0.078

Con: control; AC: AlgoClay (450 mg/kg/feed); FB: fumonisins (5.29 mg FB1 + FB2/kg/feed); FB + AC: fumonisins (5.75 mg FB1 + FB2/kg) plus AlgoClay (450 mg/kg). Results are expressed in ng FB1/g and in nmol SL/g of the liver as mean ± SD, n = 5 for groups Con and AC, and n = 6 for groups FB and FB + AC. ANOVA was used to assess the difference between groups. Statistically different groups are identified by different letters (*p* < 0.05).

**Table 4:** Effects of feeding diets containing fumonisins (FB) and AlgoClay (AC) alone or in mixtures (FB + AC) for 4, 9, and 14 days on the FB1 concentration, Sa:So ratio, C16, Sum of C22-24, Sum, and C22-24:C16 ratio from different classes of sphingolipids on the kidney of pigs.

Analytes	Con	AC 4 d	AC 9 d	AC 14 d	FB 4 d	FB 9 d	FB 14 d	FB + AC 4 d	FB + AC 9 d	FB + AC 14 d	*p*-Value
FB1 (ng/g of kidney)	ND	ND	ND	ND	ND	ND	0.50 ± 0.27 ^a^	ND	ND	0.26 ± 0.1 ^b^	0.047
Sa:So	0.1 ± 0.03 ^bc^	0.09 ± 0.01 ^c^	0.09 ± 0.02 ^c^	0.12 ± 0.02 ^abc^	0.13 ± 0.03 ^ab^	0.1 ± 0.03 ^bc^	0.16 ± 0.05 _a_	0.09 ± 0.01 ^c^	0.09 ± 0.01 ^c^	0.13 ± 0.04 ^ab^	0.002
18:1/16:0	120 ± 24 ^a^	99 ± 13 ^a^	108 ± 25 ^a^	94 ± 9 ^a^	101 ± 20 ^a^	104 ± 9 ^a^	71 ± 12 ^b^	101 ± 16 ^a^	95 ± 16 ^a^	89 ± 17 ^a^	0.003
18:2/16:0	2.86 ± 0.69	2.73 ± 0.41	3.1 ± 0.82	2.66 ± 0.44	2.71 ± 0.45	2.66 ± 0.53	2 ± 0.38	2.48 ± 0.43	2.48 ± 0.45	2.32 ± 0.5	0.060
Hex18:1/16:0	3.8 ± 1.31	4.08 ± 0.64	3.52 ± 0.55	3.23 ± 0.7	3.25 ± 0.25	3.8 ± 1.13	2.77 ± 0.63	3.32 ± 0.97	3.38 ± 0.79	3.89 ± 1.03	0.304
SM18:1/16:0	287 ± 47	274 ± 25	257 ± 19	237 ± 8	235 ± 30	245 ± 16	236 ± 21	269 ± 56	259 ± 20	251 ± 13	0.078
Sum C22-24 Cer	210 ± 35 ^a^	180 ± 24 ^abc^	184 ± 56 ^bc^	183 ± 31 ^bc^	170 ± 34 ^c^	213 ± 15 ^a^	149 ± 17 ^c^	174 ± 17 ^bc^	207 ± 33 ^a^	176 ± 15 ^bc^	<0.001
Sum C22-24 d18:2-Cer	9.44 ± 3.68	7.76 ± 0.2	8.2 ± 1.67	7.32 ± 1.13	7.42 ± 1.86	8.53 ± 1.07	6.07 ± 1.15	7.49 ± 1.58	8.54 ± 1.62	7.44 ± 1.67	0.104
Sum C22-24 HexCer	36.1 ± 13.5	37.5 ± 11.2	31.1 ± 6.1	31.9 ± 9.4	27.7 ± 3.3	30.7 ± 6.1	25.5 ± 7.2	28.2 ± 7.8	30.6 ± 7.0	35.7 ± 6.0	0.294
Sum C22-24 SM	1584 ± 303	1535 ± 159	1353 ± 183	1281 ± 88	1281 ± 235	1428 ± 131	1455 ± 129	1418 ± 269	1502 ± 197	1462 ± 99	0.148
Sum Cer	463 ± 83 ^a^	386 ± 47 ^ab^	397 ± 110 ^ab^	383 ± 55 ^ab^	367 ± 67 ^ab^	432 ± 33 ^a^	308 ± 44 ^b^	372 ± 48 ^ab^	419 ± 65 ^ab^	366 ± 45 ^ab^	0.023
Sum d18:2-Cer	15.2 ± 5	12.9 ± 0.5	14 ± 2.8	12.4 ± 1.6	12.7 ± 3.4	13.6 ± 1.7	10.1 ± 1.9	12.6 ± 2.4	13.8 ± 2.3	12.2 ± 2.5	0.106
Sum HexCer	43.5 ± 16.2	45.1 ± 12.4	37.4 ± 6.9	37.8 ± 10.6	33.4 ± 4	37.4 ± 7.4	30.5 ± 8.5	34.2 ± 9.6	36.6 ± 8.6	42.9 ± 8.2	0.276
Sum SM	2625 ± 438	2513 ± 203	2229 ± 297	2155 ± 113	2119 ± 332	2324 ± 181	2368 ± 166	2371 ± 443	2465 ± 227	2371 ± 114	0.157
Ratio C22-24:C16 Cer	1.78 ± 0.21 ^cd^	1.82 ± 0.12 ^bcd^	1.69 ± 0.16 ^d^	1.94 ± 0.25 ^abcd^	1.69 ± 0.1 ^d^	2.05 ± 0.12 ^abc^	2.12 ± 0.28 ^ab^	1.74 ± 0.15 ^cd^	2.18 ± 0.2 ^a^	2 ± 0.22 ^abcd^	<0.001
Ratio C22-24:C16 d18:2-Cer	3.3 ± 0.95	2.9 ± 0.51	2.69 ± 0.25	2.78 ± 0.44	2.71 ± 0.39	3.26 ± 0.4	3.03 ± 0.14	3.01 ± 0.36	3.48 ± 0.43	3.21 ± 0.26	0.042
Ratio C22-24:C16 HexCer	9.43 ± 1.08	9.1 ± 1.61	8.83 ± 0.88	9.8 ± 1.2	8.5 ± 0.68	8.4 ± 1.81	9.16 ± 1.39	8.56 ± 0.98	9.09 ± 0.52	9.36 ± 1.15	0.625
Ratio C22-24:C16 SM	5.4 ± 0.3 ^ab^	5.48 ± 0.44 ^ab^	5.13 ± 0.39 ^b^	5.31 ± 0.36 ^b^	5.31 ± 0.39 ^b^	5.73 ± 0.35 ^ab^	6.07 ± 0.41 ^a^	5.19 ± 0.28 ^b^	5.68 ± 0.48 ^ab^	5.74 ± 0.47 ^ab^	0.005

Con: control; AC: AlgoClay (450 mg/kg/feed); FB: fumonisins (5.29 mg FB1 + FB2/kg/feed); FB + AC: fumonisins (5.75 mg FB1 + FB2/kg) plus AlgoClay (450 mg/kg). Results are expressed in ng FB1/g and in nmol SL/g of the kidney as mean ± SD, n = 5 for groups Con and AC, and n = 6 for groups FB and FB + AC. ANOVA was used to assess the difference between groups. Statistically different groups are identified by different letters (*p* < 0.05).

**Table 5:** Effects of feeding diets containing fumonisins (FB) and AlgoClay (AC) alone or in mixtures (FB + AC) for 4, 9, and 14 days on the C16, Sum of C22-24, Sum, and C22-24:C16 ratio from different classes of sphingolipids on the lungs of pigs.

Analytes	Con	AC 4 d	AC 9 d	AC 14 d	FB 4 d	FB 9 d	FB 14 d	FB + AC 4 d	FB + AC 9 d	FB + AC 14 d	*p*-Value
Sa:So	0.15 ± 0.02 ^c^	0.14 ± 0.02 ^c^	0.2 ± 0.05 ^abc^	0.23 ± 0.09 ^abc^	0.21 ± 0.06 ^abc^	0.16 ± 0.02 ^bc^	0.29 ± 0.06 ^a^	0.16 ± 0.02 ^bc^	0.28 ± 0.09 ^a^	0.24 ± 0.07 ^ab^	<0.001
Sa1P:So1P	0.2 ± 0.04 ^bc^	0.2 ± 0.03 ^bc^	0.22 ± 0.06 ^bc^	0.26 ± 0.06 ^abc^	0.23 ± 0.09 ^bc^	0.19 ± 0.04 ^c^	0.36 ± 0.06 ^a^	0.18 ± 0.04 ^c^	0.3 ± 0.1 ^abc^	0.32 ± 0.1 ^ab^	<0.001
18:1/16:0	32.9 ± 7.1 ^bc^	46.3 ± 4.5 ^a^	35.8 ± 11.6 ^b^	22.6 ± 4.9 ^cd^	29.1 ± 7.1 ^bc^	32.2 ± 4.3 ^bc^	18.6 ± 2 ^d^	27.7 ± 6.2 ^bc^	29 ± 5.1 ^bc^	25.7 ± 3.5 ^bc^	<0.001
18:2/16:0	1.35 ± 0.32 ^abc^	1.86 ± 0.27 ^a^	1.53 ± 0.37 ^ab^	1.04 ± 0.19 ^bc^	1.11 ± 0.32 ^bc^	1.28 ± 0.38 ^abc^	0.83 ± 0.22 ^c^	1.0 ± 0.25 ^bc^	1.02 ± 0.21 ^bc^	1 ± 0.29 ^bc^	<0.001
Hex18:1/16:0	5.85 ± 0.44 ^a^	5.51 ± 0.38 ^a^	4.67 ± 1.4 ^ab^	3.61 ± 0.98 ^bc^	4.24 ± 1.24 ^abc^	5.16 ± 0.76 ^ab^	3.02 ± 0.6 ^c^	5.6 ± 2.28 ^ab^	4.19 ± 0.41 ^abc^	4.61 ± 1.03 ^ab^	<0.001
SM18:1/16:0	208 ± 15 ^a^	182 ± 14 ^b^	179 ± 22 ^b^	183 ± 16 ^b^	177 ± 18 ^b^	178 ± 17 ^b^	174 ± 16 ^b^	174 ± 13 ^b^	171 ± 12 ^b^	196 ± 7 ^ab^	0.012
Sum C22-24 Cer	78.9 ± 10.0 ^b^	104 ± 10 ^a^	77 ± 25 ^b^	59.4 ± 9.6 ^b^	69.1 ± 16.1 ^b^	75.4 ± 9.1 ^b^	55.6 ± 7.5 ^b^	72.5 ± 12.5 ^b^	71.1 ± 15.8 ^b^	63.9 ± 10.6 ^b^	<0.001
Sum C22-24 d18:2-Cer	3.31 ± 0.73 ^ab^	4.1 ± 0.6 ^a^	3.31 ± 1.06 ^ab^	2.66 ± 0.56 ^b^	2.66 ± 0.79 ^b^	3.15 ± 0.65 ^ab^	2.24 ± 0.36 ^b^	2.63 ± 0.54 ^b^	2.71 ± 0.51 ^b^	2.86 ± 0.69 ^ab^	0.007
Sum C22-24 HexCer	37.7 ± 2.8 ^ab^	39.9 ± 3.0 ^a^	32.1 ± 10.5 ^ab^	26.3 ± 6.8 ^ab^	32.9 ± 9.5 ^ab^	34.8 ± 8.2 ^ab^	25.3 ± 3.9 ^b^	36.8 ± 10.7	26.9 ± 5.5 ^ab^	32.7 ± 2.9 ^ab^	0.011
Sum C22-24 SM	960 ± 144	889 ± 47	821 ± 217	762 ± 93	841 ± 131	818 ± 121	823 ± 135	790 ± 68	783 ± 108	891 ± 49	0.267
Sum Cer	130 ± 18 ^b^	173 ± 16 ^a^	131 ± 41 ^b^	95 ± 15 ^bc^	114 ± 25 ^bc^	125 ± 14 ^b^	87 ± 11 ^c^	116 ± 19 ^bc^	115 ± 23 ^bc^	104 ± 16 ^bc^	<0.001
Sum d18:2-Cer	5.39 ± 1.17 ^abc^	6.86 ± 0.93 ^a^	5.65 ± 1.76 ^ab^	4.19 ± 0.77 ^bc^	4.31 ± 1.29 ^bc^	5.1 ± 1.09 ^abc^	3.48 ± 0.59 ^c^	4.13 ± 0.8 ^bc^	4.31 ± 0.69 ^bc^	4.43 ± 1.08 ^bc^	<0.001
Sum HexCer	45.1 ± 3.1 ^ab^	47.3 ± 3.3 ^a^	38.2 ± 12.5 ^ab^	30.9 ± 7.8 ^b^	38.5 ± 11 ^ab^	41.6 ± 9.2 ^ab^	29.4 ± 4.5 ^b^	44.2 ± 13 ^ab^	32.2 ± 6.1 ^ab^	38.7 ± 4.3 ^ab^	0.006
Sum SM	1530 ± 214	1377 ± 75	1290 ± 315	1229 ± 156	1310 ± 189	1292 ± 180	1292 ± 199	1232 ± 96	1227 ± 165	1406 ± 70	0.165
Ratio C22-24:C16 Cer	2.47 ± 0.43 ^ab^	2.25 ± 0.2 ^b^	2.17 ± 0.36 ^b^	2.65 ± 0.32 ^ab^	2.42 ± 0.42 ^ab^	2.35 ± 0.16 ^ab^	2.98 ± 0.12 ^a^	2.68 ± 0.43 ^ab^	2.45 ± 0.36 ^ab^	2.48 ± 0.15 ^ab^	0.009
Ratio C22-24:C16 d18:2-Cer	2.49 ± 0.49 ^ab^	2.22 ± 0.22 ^ab^	2.15 ± 0.32 ^b^	2.57 ± 0.24 ^ab^	2.42 ± 0.27 ^ab^	2.56 ± 0.47 ^ab^	2.78 ± 0.38 ^ab^	2.69 ± 0.46 ^ab^	2.7 ± 0.5 ^ab^	2.91 ± 0.3 ^a^	0.035
Ratio C22-24:C16 HexCer	6.46 ± 0.31 ^ab^	7.25 ± 0.62 ^ab^	6.83 ± 0.57 ^ab^	7.33 ± 0.9 ^ab^	7.8 ± 1.27 ^ab^	6.71 ± 0.88 ^ab^	8.48 ± 0.96 ^a^	6.9 ± 1.49 ^ab^	6.38 ± 0.87 ^b^	7.27 ± 0.98 ^ab^	0.028
Ratio C22-24:C16 SM	4.51 ± 0.39	4.81 ± 0.38	4.44 ± 0.6	4.07 ± 0.23	4.65 ± 0.38	4.5 ± 0.37	4.64 ± 0.51	4.48 ± 0.24	4.49 ± 0.39	4.48 ± 0.18	0.274

Con: control; AC: AlgoClay (450 mg/kg/feed); FB: fumonisins (5.29 mg FB1 + FB2/kg/feed); FB + AC: fumonisins (5.75 mg FB1 + FB2/kg) plus AlgoClay (450 mg/kg). Results are expressed in nmol/g of the lung as mean ± SD, n = 5 for groups Con and AC, and n = 6 for groups FB and FB + AC. ANOVA was used to assess the difference between groups. Statistically different groups are identified by different letters (*p* < 0.05).

**Table 6:** Main effects of feeding diets containing fumonisins (FB) and fumonisins + AlgoClay (FB + AC) for 4, 9, and 14 days, and a review of the literature data on the effects of fumonisins on biomarkers in pigs.

Group Dose ^1^	Time (Number) ^2^	Tissues	Effect	Ref
FB: 5.29 FB + AC: 5.75	4, 9, 14 d (n = 6)	Liver, kidney, lung, plasma	FB: FB1 dosed in liver at 4, 9, 14 d, and in kidney at 14 d Sa:So increased at 9 and 14 d in liver and at 14 d in kidney and lung, Sa1P:So1P increased at 14 d in lung and plasma C16-Cer decreased at 14 d in liver, kidney, and lung, C22-24:C16 Cer ratio increased at 14 d in liver and kidney FB + AC: decrease of FB1 by 43% at 4 d and 29% at 14 d in liver, and 45% at 14 d in kidney Sa:So and Sa1P:So1P not different from FB C16-Cer and C22-24:C16 Cer ratio not different from unexposed control	
FB: 2 BW	0 to 24 h (n = 8)	Serum	Increase of Sa:So at 6–24 h	[35]
FB: < 1, 5, 23, 39, 101, 175	14 d (n =5)	Serum, liver, kidney, lung	Increase of Sa:So in all matrices at 23, 39, 101, and 175 mg/kg feed	[24]
FB: 1, 5, 10	15 d (n = 5)	Serum	Increase of Sa:So at 5 and 10 mg/kg feed	[41]
FB: 2 FB + FumD: 2	14, 28, 42 d (n = 35)	Serum	FB: Increase of Sa:So at 28 and 42 days FB + FumD: Sa:So not different from unexposed control	[32]
FB: 2.5 FB + clay: 2.5 FB + FUMzyme: 2.5	42 d (n = 6)	Serum, liver, kidney, lung	FB: no effect on Sa:So FB + bentonite: increased SaSo in serum and kidney FB + FUMzyme: Sa:So not different from unexposed control	[42]
FB: 3.7, 8.1, 12.2	28 d (n = 3–6)	Serum	Increase of Sa:So for 3.7 mg/kg feed only	[33]
FB: 4.4 FB + FUMzyme: 4.4 FB + FumDSB: 4.4	32 d (n = 8)	Serum	FB: Increase of Sa:So FUMzyme: Sa:So decreased by 48.8% FumDSB: Sa:So not different from FB	[36]
FB: 7.2, 14.7, 21.9, 32.7, 35.1	14, 28 d (n = 9)	Serum	Increase of Sa:So at 14 and 28 days for all doses	[34]
FB: 2 BW	9 d (n = 3)	Liver, lung	Increase of Sa:So in liver and lung Decrease of relative abundance of C16 Cer in liver, decreased of C22-24 Cer in lung	[31]
FB: 20 BW	1, 2, 3, 4 d (n = 4)	Liver, kidney, lung, pancreas	Increased of Sa:So at 1, 2, 3, and 4 days in liver, kidney, lung	[23]

^1^ Dose of fumonisins expressed as the sum of FB1 + FB2 in mg/kg feed or in mg/kg of body weight (BW). ^2^ Duration of exposure in hours (h) or in days (d) and number of pig per group (n).

## Data Availability

The original contributions presented in this study are included in the article/Supplementary Materials. Further inquiries can be directed to the corresponding author.

## Supplementary Materials

The following supporting information can be downloaded at: https://www.mdpi.com/article/10.3390/toxins17020069/s1. Figure S1. Liver and kidney weights of pigs fed different diets. Figure S2. Effects of diets containing fumonisins at the maximum recommended level, fed to pigs for 4, 9, and 14 days, on ceramides in the liver. Figure S3. Correlations between C16 ceramides and different sphingoid bases. Figure S4. Effects of diets containing fumonisins at the maximum recommended level, fed to pigs for 4, 9, and 14 days, on ceramides in the kidney. Figure S5. Effects of diets containing fumonisins at the maximum recommended level, fed to pigs for 4, 9, and 14 days, on ceramides in the lung. Table S1. Fumonisins and sphingolipids in the liver of pigs fed for 4, 9, and 14 days with diets containing fumonisins alone and in combination with AlgoClay. Table S2. Fumonisins and sphingolipids in the kidney of pigs fed for 4, 9, and 14 days with diets containing fumonisins alone and in combination with AlgoClay. Table S3. Fumonisins and sphingolipids in the lungs of pigs fed for 4, 9, and 14 days with diets containing fumonisins alone and in combination with AlgoClay. Table S4. Fumonisins and sphingolipids in the plasma of pigs fed for 4, 9, and 14 days with diets containing fumonisins alone and in combination with AlgoClay. Table S5. Levels of mycotoxins in the experimental diets. Table S6. Protocol of the animal phase. Table S7. Intra-day recovery data of the internal standards of sphingolipids measured in the liver, kidneys, lung, and plasma. Table S8. Linearity of the method measured for the sphingolipids available as standards. Table S9. Effects of feeding diets containing fumonisins (FB) alone or in mixtures (FB + AC) for 4, 9, and 14 days on the C16, Sum of C22-24, Sum, and C22-24:C16 ratio from different classes of sphingolipids on the liver, kidney, and lung of pigs.
