# Supplementary material for: Biomarkers of Fumonisin Exposure in Pigs Fed the Maximum Recommended Level in Europe
Source: Toxins (Basel). 2025 Feb 4;17(2):69. doi: 10.3390/toxins17020069 (PMC11861712; doi:10.3390/toxins17020069)
Supplement: Supplementary file 1 [file toxins-17-00069-s001.zip › Supplementary_Figures.pdf]

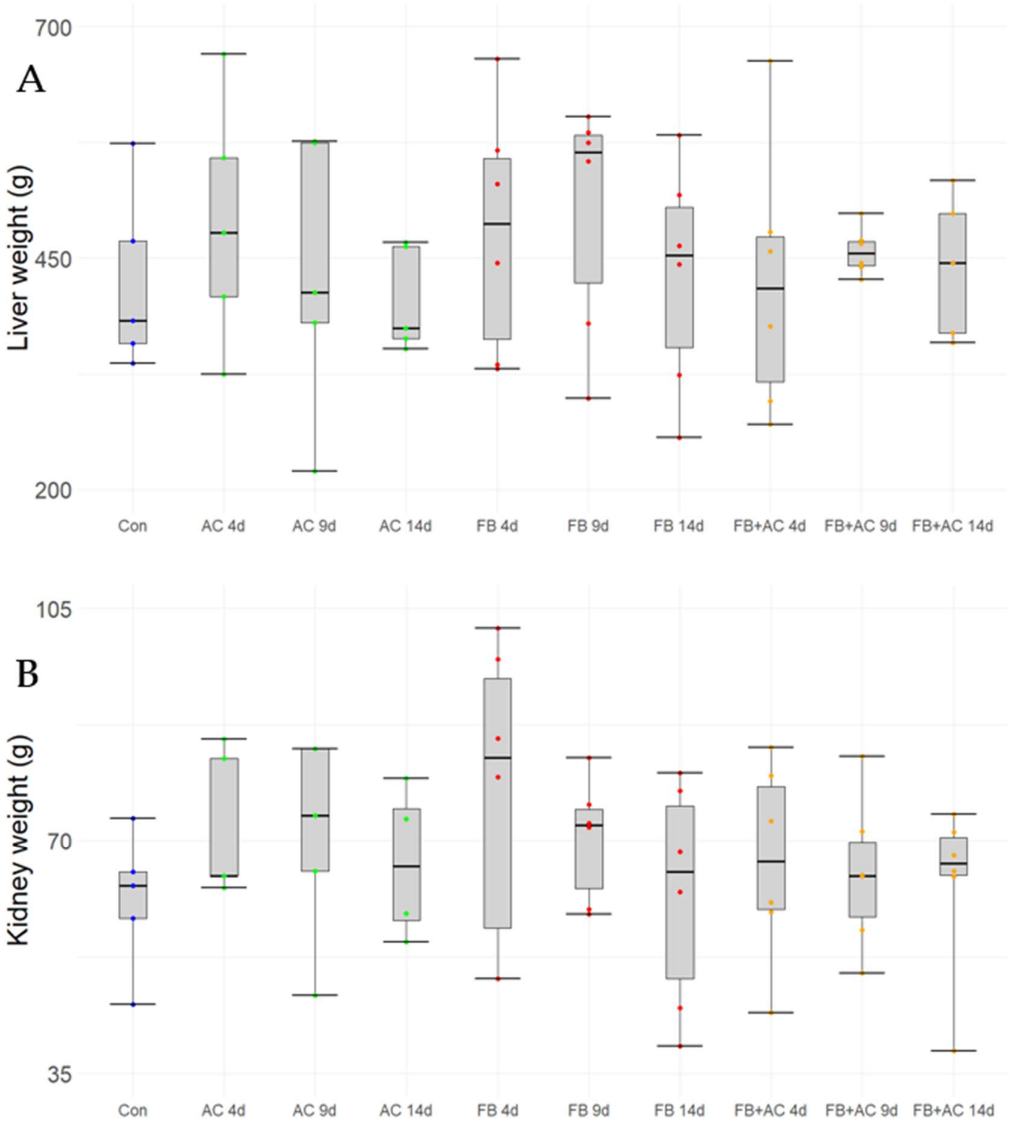

Figure S1. Liver and kidney weights of pigs fed different diets: Con (Control), FB (Fumonisins), AC (AlgoClay), and FB+AC (Fumonisins with AlgoClay). Panel (A) shows the liver weight in grams for all animals following treatment, and panel (B) shows the kidney weight in grams. There were n = 5 for the Con and AC alone groups and n = 6 for the FB alone and FB+AC groups. ANOVA was used to assess differences between groups, and no statistical difference was observed ($p > 0.05$).

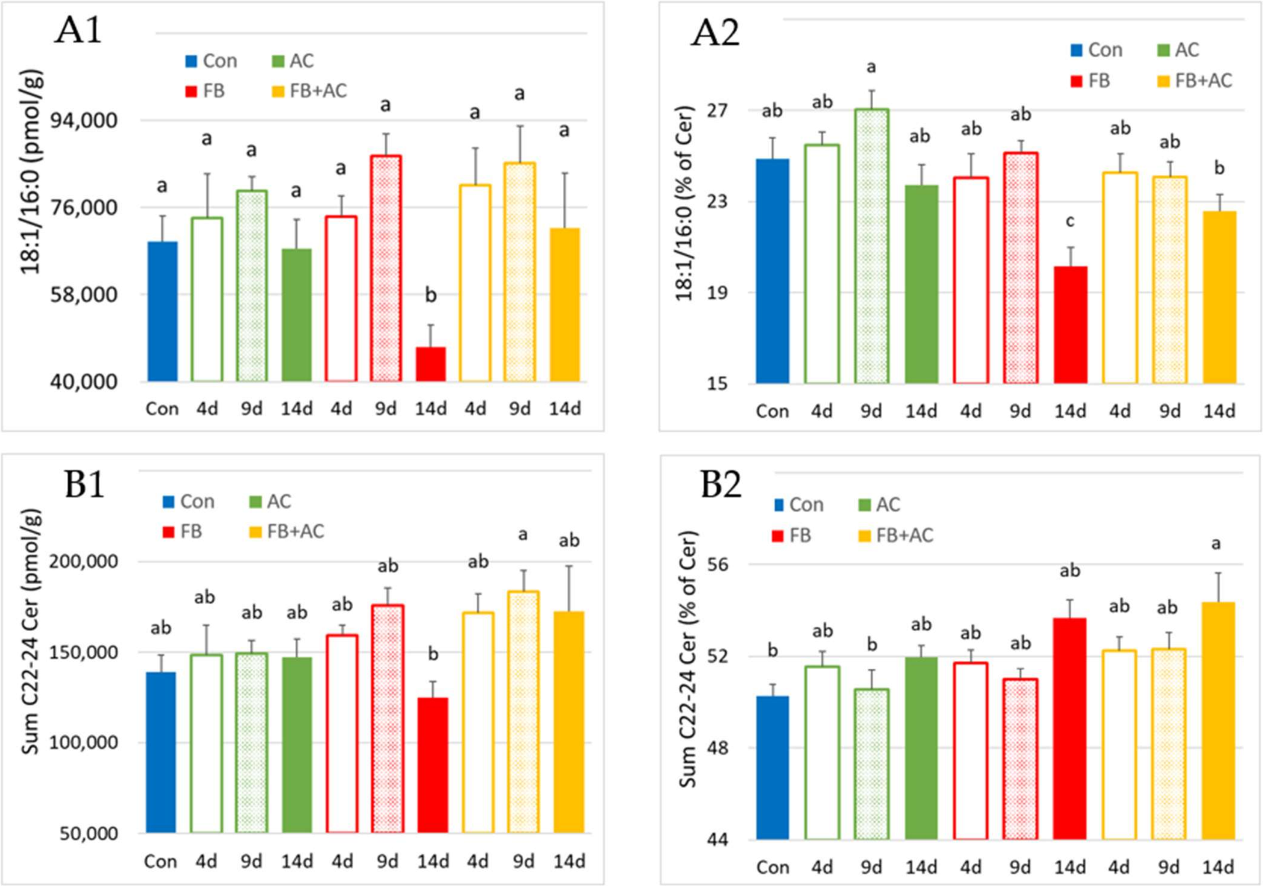

Figure S2. Effects of diets containing fumonisins at the maximum recommended level, fed to pigs for 4, 9, and 14 days, on ceramides in the liver. Panel (A) shows 18:1/16:0, with subpanel (A1) presenting the concentration and subpanel (A2) showing the percentage of 18:1-ceramides. Panel (B) shows the sum of C22 to C24 18:1-ceramids, with subpanels (B1) indicating the concentration and (B2) showing the percentage of 18:1-ceramides. The groups are labeled as Con (control), FB (fumonisins), AC (AlgoClay), and FB+AC (fumonisins plus AlgoClay). Results are expressed as the mean with standard error, with n = 5 for Con and AC, and n = 6 for FB and FB+AC. ANOVA was used to estimate differences between groups, with statistically different groups indicated by different letters ($p < 0.05$).

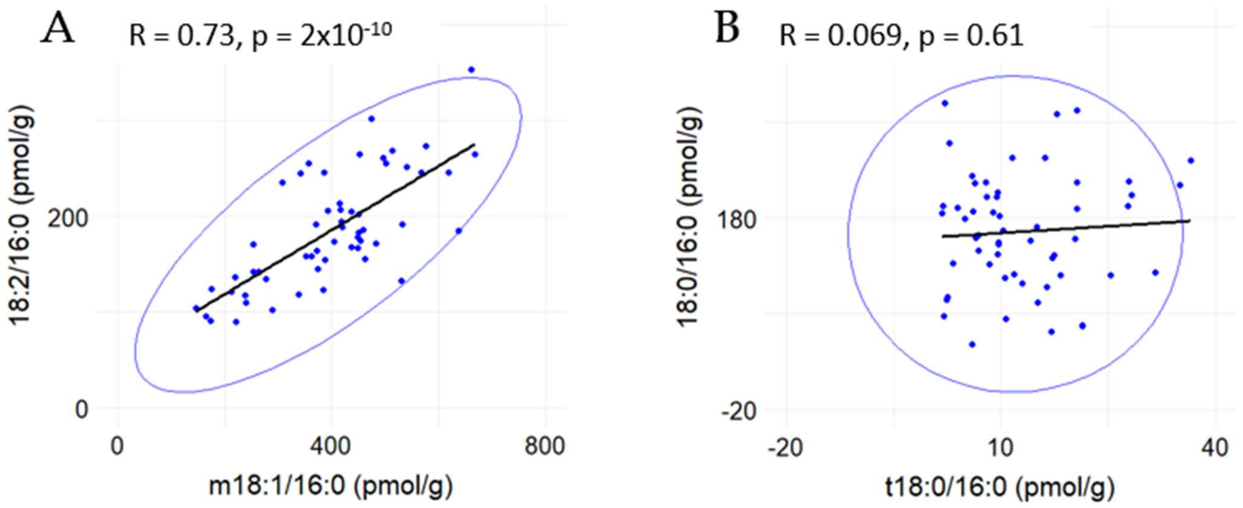

Figure S3. Correlations between C16 ceramides and different sphingoid bases. Correlation lines and confidence ellipses (0.99) were obtained from 56 pigs fed control, fumonisin, and AlgoClay diets, either alone or in combination, for 4, 9, and 14 days. Panel (A) presents the correlation between 18:2/16:0 and m18:1/16:0, while panel (B) shows the correlation between 18:0/16:0 and t18:0/16:0.

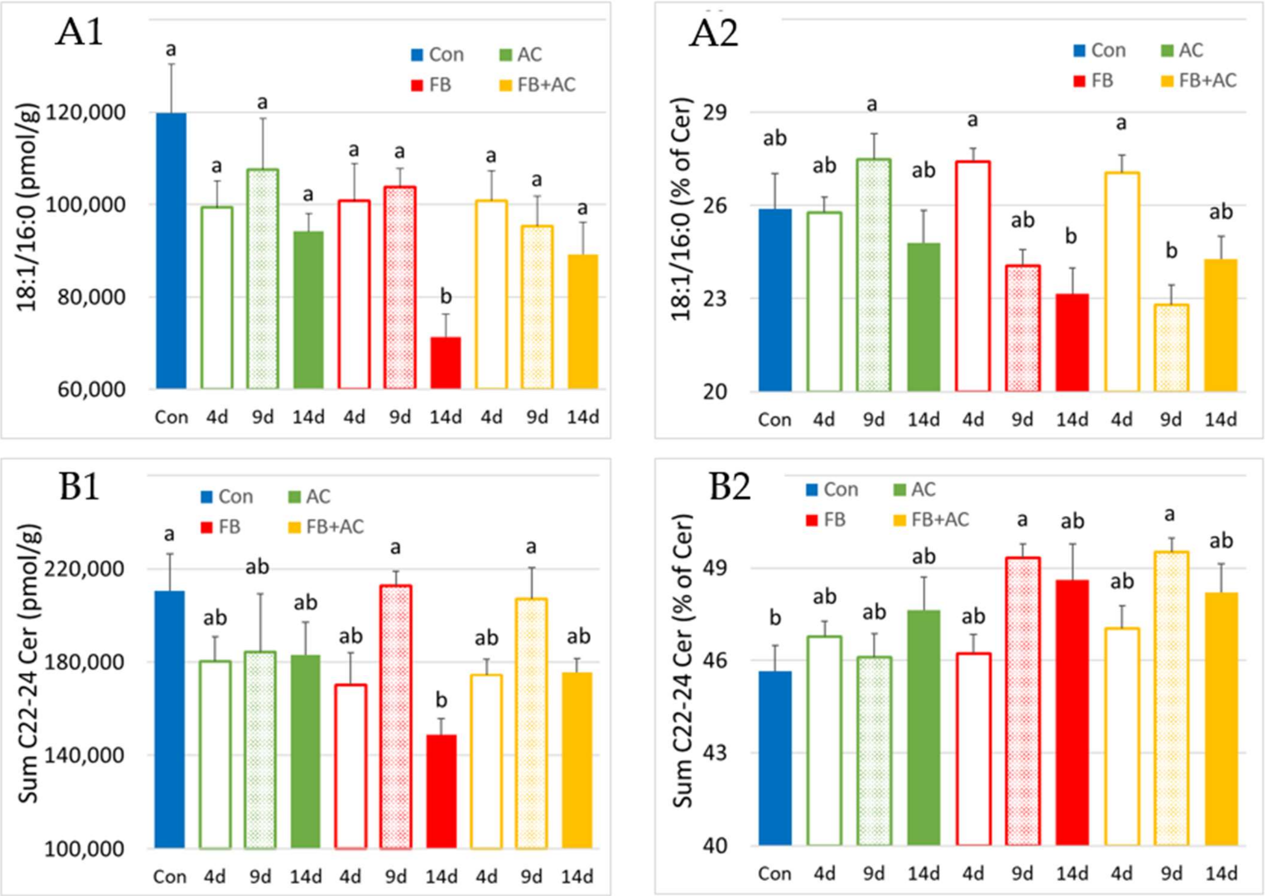

Figure S4. Effects of diets containing fumonisins at the maximum recommended level, fed to pigs for 4, 9, and 14 days, on ceramides in the kidney. Panel (A) shows 18:1/16:0, with subpanel (A1) representing the concentration and subpanel (A2) showing the percentage of 18:1-ceramides. Panel (B) illustrates the sum of C22 to C24 18:1-ceramides, with subpanels (B1) indicating the concentration and (B2) showing the percentage of 18:1-ceramides. The groups are labeled as Con (control), FB (fumonisins), AC (AlgoClay), and FB+AC (fumonisins plus AlgoClay). Results are expressed as the mean with standard error, with n = 5 for Con and AC, and n = 6 for FB and FB+AC. ANOVA was used to estimate differences between groups, with statistically different groups indicated by different letters ($p < 0.05$).

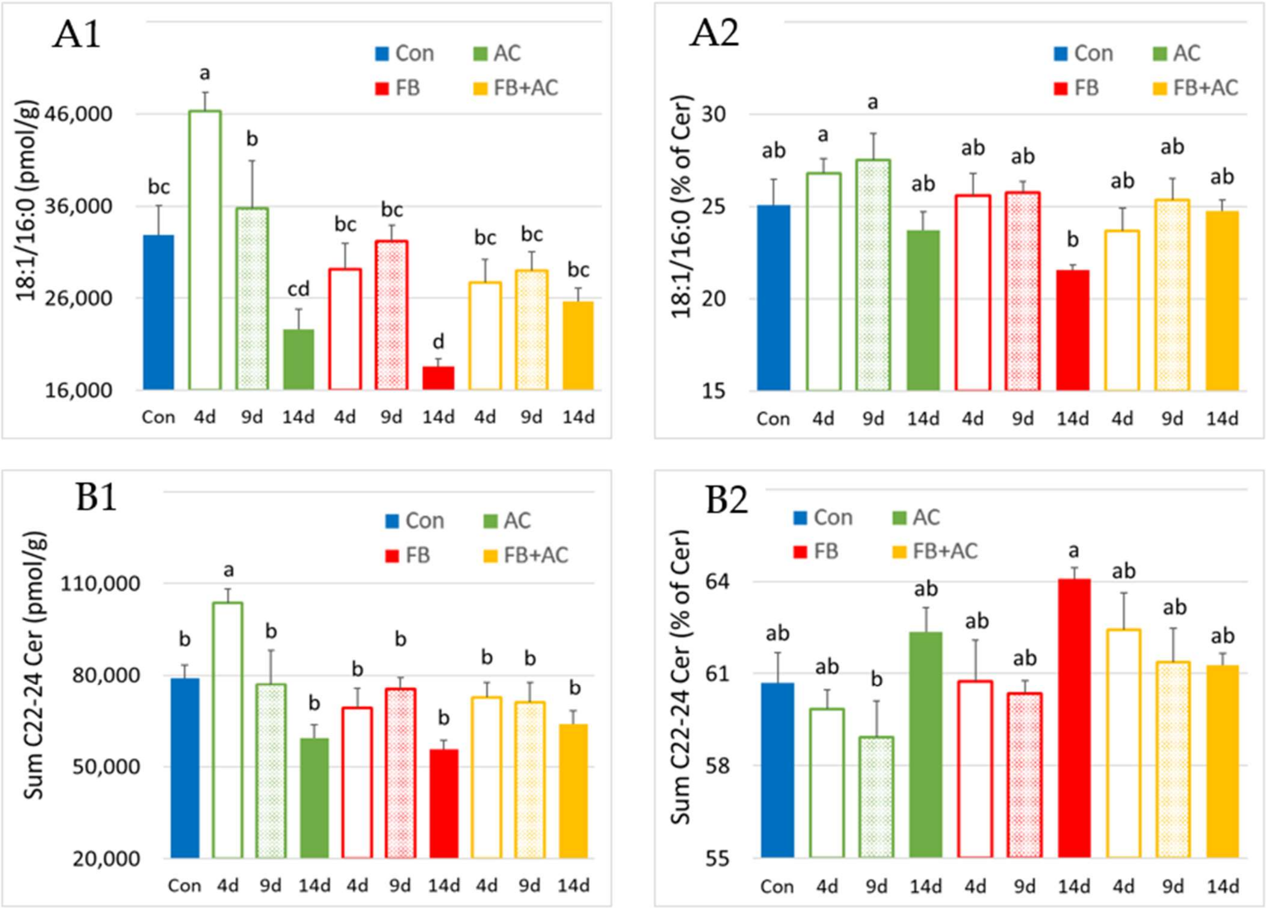

Figure S5. Effects of diets containing fumonisins at the maximum recommended level, fed to pigs for 4, 9, and 14 days, on ceramides in the lung. Panel (A) presents 18:1/16:0, with subpanel (A1) showing the concentration and subpanel (A2) representing the percentage of 18:1-ceramides. Panel (B) illustrates the sum of C22 to C24 18:1-ceramides, with subpanels (B1) indicating the concentration and (B2) showing the percentage of 18:1-ceramides. The groups are labeled as Con (control), FB (fumonisins), AC (AlgoClay), and FB+AC (fumonisins plus AlgoClay). Results are expressed as the mean with standard error, with n = 5 for Con and AC, and n = 6 for FB and FB+AC. ANOVA was used to estimate differences between groups, with statistically different groups indicated by different letters ($p < 0.05$).
